# Physical activity and healthcare utilization in France: evidence from the European Health Interview Survey (EHIS) 2014

**DOI:** 10.1186/s12889-022-13479-0

**Published:** 2022-07-15

**Authors:** Dănuț-Vasile Jemna, Mihaela David, Marc-Hubert Depret, Lydie Ancelot

**Affiliations:** 1 Faculty of Economics and Business Administration, “Alexandru Ioan Cuza” University of Iași, Iași, Romania; 2 “Gh. Zane” Institute for Economic and Social Research - Romanian Academy, Iași Branch; “Alexandru Ioan Cuza” University of Iași, Iași, Romania; 3 Centre de Recherche sur l’Intégration Economique et Financière, Institut des Risques Industriels, Assurantiels et Financiers, University of Poitiers, Poitiers, France

**Keywords:** Healthcare utilization, Physical activity, EHIS 2014, France, Recursive multivariate probit, Instrumental variables, I12, I18, C25, C26

## Abstract

**Background:**

A growing need and focus on preventing and controlling the diseases and promoting a healthier lifestyle is more evident at global, regional, and national levels. In this respect, it is well-known the positive association between physical activity and population’s health, but also its negative association with the demand of healthcare, which could lead to lower spending on healthcare systems. In France, a lack of physical activity, a high prevalence of sedentary behaviours, and a continuous deterioration of these behaviours are observed since 2006. Therefore, promoting and increasing physical activities could contribute to major societal issues. Within this context, the study aims to analyse how the use of different healthcare services are related to physical activity in a nationally representative sample of French population.

**Methods:**

The data used was retrieved from the second wave of the EHIS-ESPS 2014. The relationship between physical activity and healthcare utilization, controlled by a set of socioeconomic, demographic, and health behaviour factors, was explored both at the level of the entire population and separately for two age groups (less than 65 years, 65 years and older), employing probit and recursive multivariate probit models.

**Results:**

Our findings underline that the relation between healthcare utilization and physical activity depends on the type of healthcare services and age group. In this respect, only among adult respondents, we observe a significant negative association between physical activity and prescribed medicines consumption and day hospitalization, while preventive services use is positively related to physical activity. Common to both age groups, the positive association of physical activity with general physician services and non-prescribed medicines reveal that moderately and highly active adults and elders may be more health conscious and therefore may seek referrals to generalist and other prevention measures more frequently than their inactive counterparts. This explanation is also sustained by the negative association between physical activity and overnight hospitalization or home healthcare services.

**Conclusions:**

This study highlights the double role of physical activity on health as preventive measure and treatment and thus support the implementation of public health policies aimed at increasing the level of physical activity in French population.

**Supplementary Information:**

The online version contains supplementary material available at 10.1186/s12889-022-13479-0.

## Background

Recent statistics show that the total cost of healthcare accounted 9.6% of GDP across all the EU countries, ranging from over 11% in France, Germany, and Sweden to the lowest ratio of 5% recorded in Romania. Even if health spending grew in the previous years in line with the economy in Europe, a continuous increase of such expenses could implicate a great financial burden not only on health systems, but also on social security programs [1] and, indirectly, on society in form of reduced employment and productivity [2]. Therefore, for all EU countries, irrespective of the type of healthcare system and financing arrangement, managing the increase of health services cost is a medium- and long-term strategic objective [3]. To support this approach, it is a priority to carry out specialized studies on the population health needs, the types and frequency of the demand of health services, the factors that determine the structure and dynamics of healthcare utilization, the profile of people using the healthcare services, etc. It is equally important to assess possible means of reducing healthcare expenditure not only for ensuring access to needed care, but also for strengthening the effectiveness and the resilience of health systems [1]. In this respect, important instruments to be considered, besides cost containment policies [4] and care management strategies [5], are those related to diseases prevention and health promotion [6].

As a response to the need to prevent and control diseases and to promote a healthier lifestyle, the literature emphasizes the positive influence of physical activity on the health status of the population. It is well known that regular physical activity (1) reduces the risks for non-communicable diseases, mainly cardiovascular diseases, various types of cancer, chronic respiratory diseases and diabetes [7], (2) provides protection against future depression [8], (3) reduces stress reactions and delays the effects of various forms of dementia [9], (4) prevents the obesity, given that it is a key determinant of energy expenditure [7]. Physical activity could be considered not only as a preventive measure but also as an alternative or complementary treatment for various physical or mental health conditions. For instance, some recent studies [10–13] find consistent evidences supporting that physical activity with moderate intensity is effective in alleviating or even treating the severe symptoms of depression in affected adolescents. Interventions involving physical activity are also an accessible way of reducing the symptoms of severe anxiety or mental illness among adults, including schizophrenia-spectrum disorders, major depressive disorder, and bipolar disorder [14–18]. The effects of physical activity as an additional or stand-alone treatment are sustained in the case of other medical conditions such as: alcohol use disorder [19–23]; functional outcome after stroke [24–30]; cardiovascular disease [31]; type 2 diabetes [32]; cancer [33]. This double role of physical activity [34] reflects its negative association with demand of health services, which could lead to lower spending on healthcare systems [3, 35–37].

### Studies on the relationship between physical activity and healthcare utilization

Following our critical analysis of the literature on the relationship between physical activity and healthcare utilization, several observations are noteworthy to be mentioned. These remarks concern (1) the population for which the studies were performed, (2) the indicators used as measurements for healthcare utilization, (3) the methods and means of measuring physical activity, and (4) the control variables used in modelling the relationship between physical activity and healthcare utilization.

#### Types of population

The first observation results from the fact that most of the existing literature examines the link between physical activity and healthcare utilization just for certain segments of the population, which could depend on factors as age, gender, a particular disease, etc. A large part of such studies concentrates on older adults [36, 38–46], underlining that physical activity is strongly associated with lower usage of healthcare services. According to [38], reduced physical activity, such as walking activity, could be the most promising modifiable predictor of healthcare utilization as measured by the number of drugs and number of physician contacts over 12 months among older adults. The findings of [41, 43] indicate that being physically active might lead to beneficial results and a quicker recovery for hospitalized older adults. Analyzing only the category of older women, Silva [44] concludes that higher volumes of physical activity are significantly associated with lower usage of medications in women who are involved in a physical activity program. In this research direction, there are also strong evidence suggesting that the many benefits of physical activity for older adults extend beyond better health, improved physical function, reduced impairment, independent living, and increased quality of life to include significantly reduced healthcare costs and mortality [42–47]. Another range of studies reveals the role of regular physical activity interventions in lowering the usage of health resources and services and saving a substantial amount of healthcare expenditure among people with specific health conditions, such as asthma, cardiovascular disease, obstructive pulmonary disease, arthritis, and diabetes [42, 48–52], or those suffering from obesity problem [42, 50, 53–56]. However, it is noteworthy that the effects on healthcare utilization and costs are likely to be a result of long-time regular physical activity behaviour rather than a short-term behaviour change [56]. Of these studies, several focus on persons engaged in clinical trials fitness activity or in health program [42, 44, 45]. While their empirical evidences support that engaging in regular physical activity only involves health benefits and therefore reduced use of some health services as hospital admissions or medicine consumption, these studies have a restrictive ability to generalize to a larger population. By contrast, the literature on using representative sample from the general population is relatively limited. In this respect, a relevant, but not exhaustive enumeration of prior studies regarding the relationship between physical activity and healthcare utilization encompasses the analyses of Katzmarzyk et al. [57], Bertoldi et al. [58], Sari [59], Maresova and Vokoun [60], Rocca et al. [2], Fernandez-Navarro [61], and Kang and Xiang [37].

#### Healthcare services

The second observation concerns the dependent variables used in literature. Related to the measurement of healthcare utilization, the literature is not very explicit, but a classification of studies can be outlined. One stream focuses on obtaining an objective measure of different healthcare services through medical records kept by the family doctor, the generalist or specialist physicians [44, 45], while the second stream includes a subjective (self-related) health evaluation based on the respondents data obtained from questionnaires [2, 37–40, 42, 56, 59–61]. Within the second approach, the measures for healthcare utilization concern both service contacts [2, 39, 42, 44, 61] and volume of services [37, 38, 40, 42, 44, 45, 56, 58–60]. Usually, the literature presents four categories of healthcare utilization: medicine use, expressed in number of consumed and prescribed medication, inpatient (hospitalization and home health services), outpatient (use of generalist and specialist physicians’ services) and preventive services (dental check-up, flu shot, blood pressure check-up, cholesterol check-up, blood glucose test, immunological test).

According to literature, most of the studies concern the relationship between physical activity and one or a few healthcare categories. For instance, for the association between physical activity and medicine use there are findings to support both a significant and non-significant relationship. On the one hand, higher levels of physical activity are significantly associated with lower use of medication [27, 38, 44, 58, 61]. On the other hand, an insignificant link between physical activity and the number of medication consumed was found [27, 45]. The latest results could be attributed to the fact that these studies focused only on older adults, suggesting that other factors also should be engaged in discussions related to physical activity. Other findings from literature imply also that if people are more physically active, they will use significantly fewer inpatient services [42, 56, 59, 60] or outpatient services [38, 42, 56, 59, 60]. Having an opposite effect, physical activity appears to be a stronger predictor of all types of preventive services, emphasizing that active people may be more health conscious and thus may use precautionary measures more frequently compared to inactive persons [42]. In contrast to these results, there are studies that failed to find a significant association between physical activity and the number of days spent in hospital [38], the number of home consultations from a medical professional [45] or the number of physician’s visits [45]. In addition, the home healthcare services [45] appear not to be significantly explained by leisure time physical activity. In contrast, only few studies have analyzed the relationship between physical activity and multiple categories of healthcare utilization. For instance, Fisher et al. [39] have used both service contacts (services used versus services not used) and volume of general and specialist physician services, and hospital services, while Kang and Xiang [37] have added 10 measures of preventive services, outpatient visits, home visits, emergencies, and prescribed medicine. Their results are consistent with other studies mentioned above, but they allow to obtain a more in-depth analysis of the association between physical activity and different categories of healthcare utilization.

#### Measurements of physical activity

Another relevant remark is related to the use of different types and measurements of physical activity in relation to healthcare utilization. The physical activity is divided into four main classes, namely leisure time, household, transportation, and work. While a vast body of research focuses only on one dimension of physical activity, especially related to leisure time [2, 39, 40, 59, 61], a more narrow range of studies considers an indicator encompassing more types of physical activities [37, 56, 58, 60]. With respect to the type of physical activity, an important issue is linked to the various methods used to measure the indicator’s levels. In this matter, Dishman et al. [62], Miles [63], Sallis [64], and Sylvia et al. [65] distinguish between objective monitors (pedometers, accelerometers, heart rate monitors, armbands, and direct observations), physiological measures of energy expenditure (doubly labelled water), and self-reports (questionnaires or activity diaries). In addition, the analysis of the literature as a whole stresses the lack of studies measuring the level of physical activity by factors such as age, gender, body weight, or psychiatric and medical co-morbidities [66]. Most empirical studies evaluate and test the differences between physical activity patterns with regard to these type of factors [37, 40, 56, 61, 67–75] or explore their impact on the relation between physical activity and healthcare utilization [2, 39, 42, 45, 58, 60, 61, 76], but the authors do not integrate them into the indicator’s measuring level.

#### Other determinants of healthcare utilization

In order to gain better insight into the relationship between physical activity and healthcare utilization, most studies include a set of variables such as demographic and socioeconomic factors, health status or health behaviour. The findings adjusted for these individual characteristics reveal that involvement in physical activity still reduces the use of healthcare utilization through its relationship with chronic diseases, physical and mental health status [38, 42, 44, 56, 61], personal health practices such as smoking and drinking [44, 58], body mass index [38, 44, 58], age [2, 38, 42, 44, 56, 58], gender – with a higher effect for men [2, 38, 42, 58, 61], educational level [2, 44], economic level [2, 58], employment status [39, 60].

Beyond the use of these factors as control variables in the relationship between physical activity and healthcare utilization, there is an extensive literature on their association with the use of healthcare services [76]. It is well known that people’s health status, including inherited diseases and conditions, requires medical care. More precisely, asthma, chronic conditions, and depression are frequently related to number of physician contacts and number of drugs. In particular, prescription drugs are most strongly associated with diseases such as coronary heart disease, diabetes, hypertension, thyroid problems, osteoporosis, and heart failure [38]. Outpatient health services are more likely to be used by those who have poor to good health status, are experiencing declining health, and have chronic diseases. Meanwhile, hospitalization is more likely among those people with poor health status or having a chronic disease. However, the prevalence of these medical conditions differs by gender, age, occupational status, and other factors. The role of age is essential since, as people age, they become more susceptible to disease and disability, which implies more frequent use of various healthcare services [77]. With regard to gender, there are wide evidence that women, having higher rates of disability and self-reported fair or poor health status than men, generally use more healthcare services than their counterparts [78]. In this respect, Salganicoff et al. [79] and NCHS [80] stress that women are more likely to have primary care visits, hospitalization or emergency visit, and to receive more diagnostic services, screening services, diet and nutrition counseling than men even though men generally have higher rates of obesity and cardiovascular problems. Individual behaviours such as smoking, excessive alcohol consumption, poor diet or obesity also cause conditions that require medical attention [81]. Concerning other socioeconomic determinants of health, the literature emphasizes that higher levels of education, having economic stability, being employed, or having community safety are correlated with better health status [81].

In summary, the relatively vast body of research on the topic of this study states that interventions aimed at increasing physical activity may result in significant reductions in healthcare utilization. In addition, most of the empirical studies outlines that this potential role of physical activity is better clarify in relation to other individual characteristics. Besides identifying the determinants and assessing their association with healthcare utilization, in the end, the empirical results of such studies must be analyzed in relation to a country’s public and/or private health system and have to serve as support for other countries by sharing successes or even failures and exchanging experiences to provide inspiration for further development, refinement and implementation of effective policies.

### Physical activity in France: facts and policies

For the French population, the existing literature emphasizes a lack of physical activity and consequent sedentary behaviours, as well as a continuous degradation of these indicators in the last decades [82, 83]. Analyzing data from the ENNS study 2006–2007 and Esteban study 2014–2016, Verdot et al. [83] observe a decrease in the level of physical activity among all adult women (18–74 years old), from 63.2 to 52.7% people that are reaching the WHO recommendations on physical activity for health, while an increase is noticeable only for men (18–74 years old), from 63.2 to 70.6% [63]. The same study estimates that the prevalence of physical activity account only 50% for boys 6–17 years old and 33% for girls of the same age group. These percentages have not changed significantly between 2006 and 2016. Moreover, at the level of the EU, France is the country with the second highest prevalence of insufficient activity among school-going adolescents (86.2% in 2011 and 87.0% in 2016) [82]. For the adolescents between 11 and 14 years old it is recorded a decrease of physical activity prevalence from 38.1 to 33.7% for boys and from 23 to 20% for girls [83].

In response to this alarming reality, France was concerned to implement several national physical activity plans that include components for increasing physical activity in different sectors such as health, education, sports, transport, and workplace. In France, the integration of physical activity into public health policy dates back to the 2000s. These policies target a wide range of the population, including the people with disabilities, those suffering from chronic diseases, the elderly, the adolescents, the migrants, and other low socioeconomic groups for which specific physical activity programs are either at low cost or completely free of charge [3]. The French National Nutrition and Health Program (PNNS - Programme National Nutrition Santé), which was launched in 2001, is a public health plan that aims to improve the health status of the population by acting on one of its major determinants: nutrition. For the PNNS, nutrition is understood as the balance between food intake and physical activity. The Health Act 2004–806 also establishes certain objectives for public health policy to reduce sedentary lifestyles and increase physical activity among the French population. Another example is the accession of French specialists and institutions to the European Network for the Promotion of Health-Enhancing Physical Activity (HEPA) in 2006, one year after its launch. It should also be noted that France has taken over in various forms the guidelines formulated by The Toronto Charter for Physical Activity which was adopted in 2010 by the Global Advocacy Council of Physical Activity, International Society for Physical Activity and Health. Last but not least, in France the idea of prescribing physical activity as a treatment according to the patient’s condition, physical ability and medical risk has been formulated several times, and the idea will be implemented through the Health Act of 2016. Another successful action, called “Medicosportsanté”, is taken by the national sports federation who provides guidance on adapting sports programs for participants with chronic diseases or for the elderly. As for promoting physical activity among children and young people, an effective national intervention based on a socio-ecological approach was implemented [3]. This intervention encourages them to engage in physical activities during and outside school hours by receiving social support from parents, teachers and sports instructors. Besides the strategies countering insufficient physical activity, other recent and equally important measures to prevent diseases and promote health at the national level refer to the campaigns on tobacco and alcohol consumption and obesity among young people, raising alcohol and tobacco taxes, assessing programs and reducing work-related risks [84].

### Objective and motivation

In the EU context, all member states, including France, are involved in different projects and programs in order to promote physical activity and to evaluate its relationship with population health, and health systems. The WHO strategy for physical activity underlines as major future aims the surveillance and evaluation of policy initiatives and also the strengthening of the evidence base for physical activity and health for the EU countries [85]. Such strategy requires strengthening empirical evidence and highlighting the specificity of the relationships between physical activity, healthcare, health status, and other health risk factors in the EU context for different population groups depending on gender, age, profession or geographical area. Thereby, the implementation and the efficiency of public policies promoting physical activity and population health depend to a large extent on the health system of a country, the population structure, and a number of cultural and educational factors that can cause changes and behaviours regarding the individuals’ lifestyle and health [86].

The existing literature underlines the relevance of the association between physical activity and healthcare utilization. The increase of healthcare costs and the rising pressure on health insurance and health systems determined companies and governments to recommend physical activity as well as as complementary treatment, which in the end impacts the cost of healthcare [87]. To the best of our knowledge, in the case of French population, the research on the association between physical activity and different types of healthcare utilization is still insufficiently developed. In this regard, the outcomes of Gasparini et al. [88] and Lanhers et al. [87] should be outlined, as the authors have related the lower number of medical prescription for chronically ill patients and a lower cost of medication for type 2 diabetes in older adults to high volume of physical activities. But both studies were conducted on small and restrictive samples. Despite the generalization of their findings to the entire population, Nichèle and Yen [89] limit their study to an investigation of the role of physical activity, besides other socioeconomic characteristics and lifestyle, in the link between obesity and mental health for French adults.

Moreover, while a large body of literature provides strong evidences on the impact of physical activity and health status over healthcare utilization, only a few studies address the problem of endogeneity of these two determinants. This implies that physical activity can be itself influenced by healthcare utilization, which leads to the problem of reverse causality between the two variables. For example, as physical inactivity increases the duration of hospitalization, longer stays in hospital may also be related to the likelihood of being inactive [90]. As for the relation between healthcare utilization and health status, Bilgel and Can Karahasan [91] argue that health status is endogenous for the fact that individuals may receive healthcare and observe health status. Moreover, as Sari [59] states, it is also plausible that individuals with certain health conditions can be physically inactive and, at the same time, use more healthcare services.

In compliance with all the above underlined coordinates on the existing literature and with the EU strategy for physical activity, we aim at analyzing the association between physical activity and healthcare utilization, controlled by a set of socioeconomic and demographic factors, for a French representative sample. The contribution of this paper to the existing literature is threefold. Firstly, it provides an overall analysis of the context of healthcare utilization in relation to physical activity at the national level of France. To the best of our knowledge, no such studies have been conducted using a complex set of data provided by the European Health Interview Survey (EHIS) and the Health and Social Protection Survey (ESPS) 2014. Thus, our study provides valuable insights for policy-makers on how to improve solutions or developing programs to promote physical activity for a healthy life style in France. Secondly, following the WHO global recommendations on physical activity for health, in our paper we develop a more general measurement of physical activity that includes more components/dimensions of the indicator and also considers the age group. Hence, a more accurate classification of the population depending on the type and intensity of physical activities and age is obtained, which would be further reflected in its association with healthcare utilization. Thirdly, the methodological approach employed in the empirical analysis enables to cope with the problem of endogeneity caused by unobserved heterogeneity and possible reverse causality of healthcare utilization in relation to health status and physical activity by using instrumental variables provided by the EHIS-ESPS 2014 survey.

## Methods

### Data used

The data source for our empirical analysis is from the second wave of the EHIS 2014 carried out during the period 2013–2015 in all EU member states (in 2014 for France). In France, the EHIS 2014 has as support the Health and Social Protection Survey (ESPS) and is called EHIS-ESPS 2014, which becomes the only representative general health survey of the general population. The year 2014 represents the last wave of the field of the ESPS survey. For the 2019 wave and the following, the French version of the EHIS survey will include questions from previous ESPS on supplementary health cover. The EHIS-ESPS survey uses a main questionnaire specific to the ESPS survey, the main EHIS questionnaire applied for all EU countries, and another one on complementary French health insurance. For our empirical study, we use data from the EHIS questionnaire, but also some data from the ESPS questionnaire regarding households and socio-demographic characteristics of respondents. According to Celant et al. [92], the EHIS-ESPS survey is carried out on a sample of major beneficiaries of health insurance and the sample represents more than 92% of the population of compulsory health insurance beneficiaries. The samples of households and individuals are extracted with a probabilistic indirect sampling whose selected beneficiaries are the intermediate units. The probability of inclusion of a household, and therefore of each of its members, is equal to the probability that at least one of its members is sampled. The weight calculation method best suited to this indirect sampling method is the weight share method [92]. In order to ensure the representativeness of the sample of persons over 15 years of age in the EHIS survey, its calibration was performed using the variables: age, sex, household size, geographical area, type of insurance, additional insurance, level of education [92]. In 2014, a total of 21,101 self-administered questionnaires for people aged 15 and over containing health questions from the EHIS have been distributed on the field: 4951 were not returned by respondents, 386 were excluded because they were filled out by unauthorized proxies or because the proxy information was not filled in, 35 have withdrawn because more than half of the questions in the EHIS survey were not completed. In the end, 15,729 booklets were retained in the EHIS-ESPS survey databases, from which 397 are partial non-responses [92]. In the case of France, Eurostat recommends a minimum effective sample size of 13,110 respondents [93]. All questionnaires have been completed during two waves, spring (January to June 2014) and autumn (September 2014 to February 2015), which makes it possible to take into account the seasonality of certain pathologies.

All the variables used to analyze the association between the individual physical activity levels and healthcare utilization are presented in Table A1 (in Appendix) and briefly described below. We also used SAS 9.4 software for data analysis.

#### Dependent variables: medicine use and healthcare services utilization

The dependent variables are assessed on the basis of the questions about individual healthcare utilization from the EHIS-ESPS survey and are listed in the Appendix (Table A1). From this group of indicators, the ones of interest are related to different measurements of medicine use and healthcare services utilization. The first type of healthcare utilization is measured by considering both self- and prescribed medications (use vs. non-use) in the previous 2 weeks. The incidence of medicines use and their dosages are not explored. Healthcare services utilization is characterized as the use of inpatient (overnight hospitalization, hospitalization during the day, and home healthcare services), outpatient (visits to generalist and specialist physician) and preventive services in the 12-month period prior to the survey. For hospital services, participants were asked to report if they had stayed overnight as a patient in a hospital in the previous year and, for those who had been hospitalized, the annual number of nights spent in a hospital was recorded. With respect to home healthcare services, respondents were asked if they had home visits from any health professional during the previous 12 months. Concerning the outpatient services, two continuous variables indicating volume of service use during the last 4 weeks and two nominal variables were used to specify the incidence of contact with both generalist and specialist physicians. For the last group of self-reported health services utilization, 6 measures of preventive services were examined: dental checkup, flu shot, blood pressure checkup, cholesterol checkup, blood glucose test, and immunological test. Based on the responses received at the questions regarding the use of these preventive services, a general variable was defined denoting if respondents are cautious or not about their own health, i.e. cautious are those who use at least two preventive services suggested to be used annually, and incautious are those who do not respect the recommendations. Although both service contacts (services used versus services not used) and volume or frequency of using this services are important while analyzing the determinants of each type of healthcare utilization, in this paper the incidence of using different health services is not explored.

#### Main independent variable: physical activity

Physical activity is the main independent factor of interest in our analysis. The basis for measuring the indicator levels consists of respondents’ answers to a set of questions on the frequency and time spent on physical activities related to transport and leisure during a typical week. For the transport domain, walking and cycling are considered physical activities, and respondents provided information on the number of days per week and the duration in minutes per day spent on these activities. The duration per day is registered in minutes in six ranges: less than 10; 10–29; 30–59; 60–120; 120–180; 180 and more minutes. Regarding leisure time, respondents provided data on sports practice, recorded as number of days per week and number of minutes per day, while for muscle building exercises, only the number of days per week was taken in consideration.

For the measurement of physical activity in the population over 15 years of age, the literature presents several methods such as IPAQ-SF [94] or EHIS-PAQ [95]. The major difference between the two methodologies is that IPAQ-SF proposes a total physical activity level based on MET (metabolic equivalent) computed by summing the duration (in minutes) and frequency (days) of walking, moderate intensity and vigorous intensity activities, while EHIS-PAQ evaluates how far the population is physically active in specific public health relevant settings (work, transport, leisure). However, Finger et al. [95] argue that although the EHIS-PAQ is not designed to construct a total physical activity index, MET calculations are possible for transport-related and leisure-time domain. Nevertheless, in the computation of physical activity both IPAQ-SF and EHIS-PAQ tools do not take into consideration the age of respondents, which is a very important factor knowing that the ability to exert physical effort is different from one age group to another. Therefore, in this study we aim at measuring physical activity by addressing this limit.

In this context, age is stratified into three intervals as defined in the ANSES Report [96]: 15–17 years, 18–65 years, and more than 65 years. Consequently, for the assessment of physical activity levels, we propose several steps that imply combining the IPAQ methodology [94] and the WHO global recommendations on physical activity for health [96]. All these steps are presented in the Appendix (Tables A3 to A7). We start from the MET values and formula for the computation of MET-minutes spent on physical activity per week, taking into account only transportation and leisure-time domains (Tables A3 and A4). Regardless of the context or the domain in which physical activity is practiced (work, transport, domestic activities or leisure), it can take different forms. Hence, in compliance with the ANSES Report [96], we considered the types of activities according to the physiological functions required: cardio respiratory, muscular, relaxation and balance. Moreover, in line with the international recommendations [7], the classification on age group with regard to these functions is detailed in Table A5. Nonetheless, these recommendations emphasize that physical activities should vary not only in their types, but also in their intensities. The different types of physical activities can be sorted in five main categories (sedentary, light, moderate, high, and very high) according to their estimated intensity in MET (Table A6). These criteria enabled us to define walking as an activity of moderate intensity and the other three, namely riding a bike, making sports and muscle building exercises, as high intensity activities. Finally, combining the above mentioned coordinates, physical activity is defined considering each group of age. Thus, the respondents was categorized as active, moderately active, or low active, according to each age group and the reported frequency and duration associated with all the leisure-time and transport-related physical activity (Table A7).

#### Control variables

The control variables included in the analysis are divided into three major topics such as (1) individual characteristics, (2) health status characteristics, and (3) health behavioural factors.

1. The individual characteristics correspond to demographic and socioeconomic determinants such as gender, age, education level, marital status; employment status, health insurance status, income level. The marital status was sorted into two groups according to several studies [39, 59]: married; unmarried (including four categories of the initial variable, namely single, divorced, widower, and concubine). The education level was measured by means of the last degree obtained by respondents and according to the ISCED 2011 into three categories (low education; medium education; high education), to which we added a fourth one (student) for those who, at the time of the survey, according to their socio-professional category, were still involved in studies. Employment status is expressed as a dichotomous variable indicating if respondents are employed or unemployed (including respondents in school or not attending school and those without job). Concerning the health insurance status, the respondents were divided into three categories: those without health insurance; those having a complementary health insurance; and those having a private health insurance. Finally, the level of income was defined with the help of the median value of the individual’s income (household income divided by the number of members), which was 1500 euros according to the data provided by the ESPS household survey.
2. The health status section encompasses various dimensions of health status and health-related activity conditions or limitations: chronic conditions, long-term health conditions, depression, and limitations. Following the SF-36 scoring methodology [97], all these factors were used to build a general health status index, which included the respondents into four categories: poor health, fair health, moderate health, and good health. The classification of respondents into the four groups was based on the quartiles obtained from the health score.
3. The last topic consists of different individual and environmental determinants describing four different health behaviours represented by several behavioural predisposing factors of health: height and weight, which were used as measures for the Body Mass Index (BMI), which divides the sample into normal weight, overweight, and obese categories according to WHO recommendations [98]; smoking categorized in light or heavy daily smoker, occasional smoker, and no-smoker; alcohol consumption expressed by an aggregated variable indicating the *alcohol profile* that, according to Maresova and Vokoun [60], divides individuals into risk (one-time risk consumer, chronic and dependent consumer) and risk-free consumption (no-consumer, safe consumer); fruits and vegetables consumption, which were used to define the participants’ nutritional behaviour following the French ANSES’s recommendations [96] of consuming at least 5 fruits and vegetables every day. Thereby, the new variable divides the respondents in two groups: those who respect these preventive recommendations and those who do not.

### Empirical strategy

This section explains the econometric framework used to assess the relationship between physical activity and the dependent dichotomous variables indicating the use or non-use of each type of medicine and healthcare services. Given the discrete nature of our measures, we estimate the following regression model:

1 $$HCU=\alpha +{\beta}_j PA+{\gamma}^{\prime }X+\varepsilon$$

where *HCU* stands for healthcare utilization and refers to one of the dependent variables described above; physical activity (*PA*) is our variable of interest that takes the value 0 for low physically active group, the value 1 for a moderate level of physical activity, and the value 2 if the individuals are high physically active; *X* is a vector of control variables, which were introduced in the previous section; and *ε* is the residual component. For the significance of the regression coefficients, four significance thresholds are considered: 0.1, 1, 5, and 10%.

We are interested in the estimation of parameters *β*_*j*_ from Eq. (1), which is provided by means of multiple probit regression techniques [99]. These coefficients refer to the change of the normal Z-score variable or the probit index for a one-unit change of the numerical independent variable. In the case of a nominal variable, the regression coefficient estimates the difference between the probit index for a given group and the reference group. Important for interpretation is the sign of the coefficients. Thus, a positive coefficient indicates that compared to the reference group, the group indicated by the independent variable has a higher probability of realizing the event observed by the dependent variable.

However, the potential reverse causality between physical activity and healthcare utilization may lead to endogeneity and, implicitly, to biased estimates [90]. Furthermore, physical activity may affect healthcare utilization through its relationship with overall health of individuals [41, 53, 59]. In order to account for variations in health that may affect both the level of physical activity and healthcare utilization, this paper uses a general health status index. At the same time, healthcare utilization may determine the health status of individuals [91]. Therefore, to tackle the potential endogeneity issue of both physical activity and health status, we apply recursive multivariate probit model that involves the use of instrumental variables estimation. The instruments should not be directly related to healthcare utilization, except for their link to physical activity and overall health status [100, 101]. For health status, the instrumental variables are represented by the education level of respondents’ parents [102] and their attitude towards the future [102], while for physical activity, the membership to an association or a sport club was considered as a proxy for the distance to physical fitness and sports facilities [101].

Thus, using recursive multivariate probit model, Eq. (1) becomes as follows [99, 103]:

2 $${HCU}^{\ast }=\alpha +{\beta}_j{PA}^{\ast }+{\delta}_j{HSI}^{\ast }+{\gamma}^{\prime }X+{\varepsilon}^{\ast }$$

$${PA}^{\ast }=\theta +{\delta}_j HSI+{\gamma}^{\prime }X+{\delta}^{\prime }Y+u$$

$${HSI}^{\ast }=\phi +{\beta}_j PA+{\gamma}^{\prime }X+{\delta}^{\prime }Z+\nu,$$

where health status index (*HSI*) takes the values 0 (poor health), 1 (moderate), 2 (good health), and 3 (very good health) and physical activity (PA) is coded with 0 (low physically active), 1 (moderate physically active), 2 (high physically active); *Y* represents the vector of instrumental variables for physical activity and *Z* is the vector of instrumental variables for health status index, which are excluded from Eq. (1) and assumed to be uncorrelated with the residual terms, *u* and *ν*, but correlated with healthcare utilization (*HCU*) only through its link to on being moderate or high physically active and being in a state of moderate, good, or very good health; *X* is the vector of covariates used in the previous equation; and *ε*^∗^ is the residual component.

Next, different robustness and sensitivity checks to test the two estimation approaches (probit and recursive multivariate probit) are performed. In a multivariate probit model, the likelihood ratio (LR) test is used to test exogeneity, by comparing the log-likelihood of the multivariate probit model to the sum of the log-likelihoods of the marginal probit models, estimated separately [104]. These should be equal in the case of independent errors across the marginal distributions, thus the LR test compares an unrestricted model to a restricted one, considering the separate probit estimates as a multivariate probit in which all correlations are restricted to zero. The rejection of null hypothesis confirms the presence of endogeneity. Further, following Guilkey and Lance [105] and Sari and Osman [101], we perform an overidentification test to verify the validity of instrumental variables. Therefore, a likelihood ratio (LR) test is used to test the null hypothesis that the coefficients for instrumental variables are jointly zero in the healthcare utilization equation, meaning that they are not significantly associated with healthcare utilization.

Moreover, the relationship between healthcare utilization and physical activity is explored at the level of the entire population, but also separately, for two age groups, namely less than 65 years and 65 years and older. It is a fact of life that, as people age, they become more susceptible to disease and disability [106]. Therefore, this threshold of 65 years was chosen in relation to health deterioration with age and in compliance with the work of Verdot et al. [83]. In addition, as Fisher et al. [39] point out, there is considerable heterogeneity within the adult population relative to physical activity, health status, and healthcare utilization. Thereby, classifying the respondents in groups by age, and not treating them as one homogeneous group, enables a more in-depth analysis of the response of healthcare utilization to different levels of physical activity.

## Results

### Descriptive statistics

The first part of this section presents the sample distribution for the dependent variables on the two major age groups and stratified by physical activity levels (Table 1). Similarly, the sample distribution for the control variables is presented in the Appendix (Table A2).

**Table 1:** Healthcare utilization, both for the entire population and stratified by age group and physical activity level

HEALTHCARE UTILIZATION		TOTAL (%)			***<*** 65 YEARS (%)			***≥*** 65 YEARS (%)		
		Physical Activity			Physical Activity			Physical Activity		
		Low	Moderate	High	Low	Moderate	High	Low	Moderate	High
Non-prescribed medicines	*No*	78.53	71.98	72.18	76.85	70.64	69.86	82.86	80.51	77.46
	*Yes*	21.47	28.02	27.82	23.15	29.36	30.14	17.14	19.49	22.54
Prescribed medicines	*No*	41.52	46.59	50.96	53.66	53.90	60.17	10.32	17.20	19.61
	*Yes*	58.48	53.41	49.04	46.34	46.10	39.83	89.68	82.80	80.39
Overnight hospitalization	*No*	84.07	88.85	89.75	86.69	90.39	91.10	77.32	82.65	85.16
	*Yes*	15.93	11.15	10.25	13.31	9.61	8.90	22.68	17.35	14.84
Day hospitalization	*No*	85.67	86.72	86.09	85.99	87.09	86.49	84.86	85.23	84.70
	*Yes*	14.33	13.28	13.91	14.01	13.51	12.91	15.14	14.77	15.30
Generalist physician services	*No*	13.77	11.17	12.03	17.87	12.85	13.97	3.27	4.40	5.48
	*Yes*	86.23	88.83	87.97	82.13	87.15	86.03	96.73	95.60	94.52
Specialist physician services	*No*	51.34	50.85	52.73	55.60	53.50	56.47	40.12	40.03	39.80
	*Yes*	48.66	49.15	47.27	44.40	46.50	43.53	59.88	59.97	60.20
Preventive services	*No*	46.18	47.16	50.89	54.57	57.54	60.19	15.19	15.85	17.98
	*Yes*	53.82	52.84	49.11	45.43	42.46	39.81	84.81	84.15	82.02
Home healthcare services	*No*	84.67	92.19	93.90	91.29	94.44	95.26	67.72	83.15	89.26
	*Yes*	15.33	7.81	6.10	8.71	5.56	4.74	32.28	16.85	10.74

*Source:* Authors’ computation

With the exception of specialist consultations, in all three samples, all the other healthcare services differ between each physical activity level. At the level of the entire population, across all levels of physical activity, the majority of respondents reported not using non-prescribed medications, while between 41.52% (low active group) and 50.96% (high active group) had not used prescribed medications. The proportion of respondents who had been overnight hospitalized was highest in the low active group and lowest in the high active group. Across all levels of physical activity, fewer than 15% of individuals had been hospitalized during the day in the previous 12-months period. Between 86.23% (low active group) and 88.83% (moderate active group) of individuals reported at least one contact with a generalist physician and between 47.27% (high active group) and 49.15% (moderate active group) with a specialist physician in the previous 12 months. The low active group reported using more preventive and home healthcare services than all the others, but, regardless of physical activity group, the majority of individuals reported not using home healthcare services.

In both age groups and regardless of physical activity level, the majority of respondents reported not consuming non-prescribed medicines, not being overnight or day hospitalized, not using home healthcare services, but having more visits to generalist physician. Among the respondents aged under 65 years, the consumption of prescribed medicines, overnight and day hospitalization, the use of preventive and home healthcare services decrease with the intensity of physical activity, while non-prescribed medicines consumption is associated with a higher physical activity intensity. In contrast, moderately active individuals reported using more generalist and specialist visits in comparison with their low and highly active counterparts. In the 65 years and older age group, prescribed medicines, overnight hospitalization, generalist physician services, preventive and home healthcare services are used more by low active older adults and less by the highly active group. Instead, the consumption of non-prescribed medicines and the use of specialist services are associated with higher physical activity intensity, whilst for day hospitalization the lowest proportion is observed in moderately active group. Moreover, comparing the results stratified on age groups to the ones at the level of the entire population highlights the presence of heterogeneity within age groups with respect to the link between physical activity and the use of different healthcare services.

### Main results

The results of the analysis are presented synthetically in the core text only in relation to physical activity and separately for each age group (Table 2). The full results are presented in Appendix (Tables A8-A19), but only for one specification of the regression models depending on the significance of the endogeneity test. In other words, if the test’s result indicates the presence of reverse causality, then the outcomes of recursive multivariate probit models are discussed. Otherwise, the results from multivariate probit regressions are considered.

**Table 2:** The association between physical activity and healthcare utilization, stratified by age

	Total				***<*** 65 Years Age Group				***≥*** 65 Years Age Group			
	Probit Model		Recursive Multivariate Probit Model		Probit Model		Recursive Multivariate Probit Model		Probit Model		Recursive Multivariate Probit Model	
**Non-prescribed medicines**												
Moderately active	0.1049	*p* < .001	0.0455	*p* < .1	0.0551	**.**	0.1146	*p* < .001	0.0804	p < .1	0.0676	*p* < .1
Highly active	0.1540	*p* < .001	0.0699	*p* < .1	0.1137	*p* < .001	0.1528	*p* < .001	0.1853	*p* < .01	0.1567	*p* < .1
*Test of endogeneity*	–		8.22	*p* < .001	–		7.89	*p* < .001	–		0.29	*p* < .1
*Test of overidentification*	–		2.85	*p* < .1	–		2.26	*p* < .1	–		1.88	*p* < .1
**Prescribed medicines**												
Moderately active	−0.0658	*p* < .05	− 0.0761	*p* < .01	− 0.0261	**.**	−0.0284	*p* < .1	−0.1349	*p* < .1	−0.1881	*p* < .1
Highly active	−0.1064	*p* < .001	−0.1160	*p* < .001	−0.1006	*p* < .001	−0.1439	*p* < .001	−0.1273	*p* < .1	−0.1650	*p* < .1
*Test of endogeneity*	*–*		*0.08*	*p* < .1	*–*		*2.45*	*p* < .1	*–*		*0.37*	*p* < .1
*Test of overidentification*	*–*		*1.01*	*p* < .1	*–*		*2.13*	*p* < .1	*–*		*1.77*	*p* < .1
**Overnight hospitalization**												
Moderately active	−0.1095	*p* < .01	−0.1278	*p* < .01	−0.1397	*p* < .01	−0.1521	*p* < .001	−0.0849	*p* < .1	−0.0328	*p* < .1
Highly active	−0.1296	*p* < .001	−0.1335	*p* < .001	−0.1048	*p* < .001	−0.1658	*p* < .001	−0.1124	*p* < .001	−0.0747	*p* < .1
*Test of endogeneity*	*–*		*0.10*	*p* < .1	*–*		*5.31*	*p* < .01	*–*		*0.47*	*p* < .1
*Test of overidentification*	*–*		*1.75*	*p* < .1	*–*		*0.62*	*p* < .1	*–*			
**Day hospitalization**												
Moderately active	−0.0149	*p* < .1	− 0.0178	*p* < .1	− 0.0082	*p* < .1	−0.0811	*p* < .01	0.0387	*p* < .1	0.0811	*p* < .1
Highly active	−0.0714	*p* < .1	−0.0316	*p* < .1	−0.0734	*p* < .1	−0.2783	*p* < .001	0.0484	*p* < .1	0.0643	*p* < .1
*Test of endogeneity*	*–*		*0.60*	*p* < .1	*–*		*4.52*	*p* < .01	*–*		*0.19*	*p* < .1
*Test of overidentification*	*–*		*1.70*	*p* < .1	*–*		*1.72*	*p* < .1	*–*		*0.40*	*p* < .1
**Generalist physician services**												
Moderately active	0.2090	*p* < .001	0.2346	*p* < .001	0.2264	*p* < .001	0.1802	*p* < .001	0.0178	*p* < .1	0.2615	*p* < .01
Highly active	0.2106	*p* < .001	0.2937	*p* < .001	0.2393	*p* < .001	0.1917	*p* < .001	0.0200	*p* < .1	0.2613	*p* < .01
*Test of endogeneity*	*–*		*8.18*	*p* < .001	*–*		*5.11*	*p* < .01	*–*		*6.16*	*p* < .01
*Test of overidentification*	*–*				*–*		*1.32*	*p* < .1	*–*		*0.66*	*p* < .1
**Specialist physician services**												
Moderately active	−0.0343	*p* < .1	−0.0310	*p* < .1	0.0430	*p* < .1	0.0152	*p* < .1	0.0019	*p* < .1	0.0397	*p* < .1
Highly active	−0.0415	*p* < .1	−0.1286	*p* < .001	0.0482	*p* < .1	0.0118	*p* < .1	0.0385	*p* < .1	0.1309	*p* < .1
*Test of endogeneity*	*–*		*0.93*	*p* < .1	*–*		*2.42*	*p* < .1	*–*		*0.65*	*p* < .1
*Test of overidentification*	*–*		*2.16*	*p* < .1	*–*		*0.99*	*p* < .1	*–*		0.66	*p* < .1
**Preventive services**												
Moderately active	0.0719	*p* < .01	0.0501	*p* < .1	0.0851	*p* < .01	0.0660	*p* < .05	−0.0343	*p* < .1	−0.0331	*p* < .1
Highly active	0.0826	*p* < .01	0.0657	*p* < .1	0.0880	*p* < .01	0.1185	*p* < .01	−0.0683	*p* < .1	−0.0574	*p* < .1
*Test of endogeneity*	*–*		*0.13*	*p* < .1	*–*		*0.16*	*p* < .1	*–*		*0.03*	*p* < .1
*Test of overidentification*	*–*		*3.26*	.	*–*		0.45	*p* < .1	*–*		*3.11*	*p* < .1
**Home healthcare services**												
Moderately active	−0.2152	*p* < .001	−0.2298	*p* < .001	−0.1813	*p* < .001	−0.1921	*p* < .001	−0.2137	*p* < .001	−0.2599	*p* < .001
Highly active	−0.2599	*p* < .001	−0.3151	*p* < .001	−0.2044	*p* < .001	−0.2247	*p* < .001	−0.3179	*p* < .001	−0.4213	*p* < .001
*Test of endogeneity*	*–*		*26.09*	*p* < .001	*–*		*16.46*	*p* < .001	*–*		*2.73*	*p* < .05
*Test of overidentification*	*–*		*3.08*	*p* < .1	*–*		*2.32*	*p* < .1	*–*		*0.46*	*p* < .1

(1) The table reports estimates for two approaches (probit and recursive multivariate probit) on the association between physical activity and the use of medicines and healthcare services. The coefficients indicate the difference between the probit index (Z-score) of each category of physical activity, i.e. moderately and highly physically active, and that corresponding to the reference group for physical activity, which is low physically active. (2) The relationship between physical activity and healthcare utilization is controlled by the following variables: sex of respondent; age group; education level; legal marital status; employment status; insurance; income level; BMI status; smoking; alcohol consumption risk profile; nutrition – fruits and vegetables consumption; health status index

*Source:* Authors’ computation

#### Irrespective to age group (total)

The probit modelling results pertaining to the entire population show an obvious relationship between physical activity and healthcare utilization. Regardless of the intensity of physical activity, this link is positive for the consumption of medicines without a prescription, the visits to a general practitioner, and the use of preventive services. This undoubtedly underlines a stronger tendency of those highly or moderately active towards preventive health. By contrast, physical activity is negatively associated with the consumption of prescribed drugs, the overnight hospitalization, and the use of home healthcare services. However, our findings do not support a significant link between physical activity and the hospitalization during the day or the use of a specialist practitioner services.

To complete this first step of analysis, we further account for the endogeneity of both physical activity and health status in the initial probit model by using instrumental variables in the context of recursive multivariate probit regressions. Based on the LR statistics results, the reverse causality is confirmed for the use of only three healthcare services, namely non-prescribed medicines’ consumption, visits to the generalist physician, and home healthcare services, in relation to physical activity and health status. After solving the endogeneity problem, the estimates are consistent in sign and a higher magnitude of the coefficients corresponding to the relationship between these particular healthcare services is obtained. However, when the consumption of non-prescribed drugs is considered, whilst probit regression indicates positive and statistically significant results, the recursive multivariate probit results indicate also positive, but insignificant coefficients. These findings, therefore, highlight that the choice of the empirical strategy is important and requires further robustness checks. In this regard, we perform an overidentification test of a null hypothesis that the coefficients for the instrumental variables are jointly zero in the equations of the three healthcare utilization services. As shown in Tables (A8)–(A10), the *p*-values are high enough not to reject the null hypothesis, suggesting that these instrumental variables are valid and properly excluded from the healthcare utilization equations. With respect to substance, this suggests, on the one hand, that the respondents’ likelihood of being healthier is positively related to parents’ education and the fact of being concerned about the future, which in the end is reflected in a higher negative association between health status and the use of non-prescribed medicines, the visits to generalist, and the use of home healthcare services. On the other hand, the individuals implicated in social activities are more likely to be moderately and highly physically active, which also affects the link between physical activity and the use of healthcare services.

With respect to control variables, besides the respondents’ health status, both socioeconomic characteristics and health behaviour were consistently associated with the use of all healthcare services (Tables A8-A11). Nevertheless, irrespective to model’s specifications and the type of healthcare utilization, age group and health status index prevailed. On the one hand, the individuals with a better health status were less likely to use any of the healthcare services compared to the ones in poor health. On the other hand, the results show that people were more likely to use health services with higher age. Therefore, the considerable heterogeneity within the subgroups of population relative to physical activity and healthcare utilization justifies the further analysis by age groups. The findings on the relationship between physical activity and healthcare utilization stratified by age group are summarized in Tables (2)–(3) and presented in detail in Appendix (Tables A12-A19).

#### <65 years age group

After adjusting for other factors related to healthcare utilization, but without controlling for possible endogeneity, the results of the regression analysis pertaining to the < 65 years age group (Table 2) show an increased probability of using non-prescribed medicine, general physician’s services, or preventive services among moderate and high active individuals compared with their low active counterparts. In contrast, moderate and high physically active respondents were less likely to be higher consumers of prescribed drugs, to have had an overnight hospitalization or to call on home healthcare services in the previous 12 months, but they were as likely as the low active individuals to have had a day hospitalization or to use specialist physician’s services during the last year. These results are very similar (both in coefficients’ magnitude and sign) to those obtained for the entire population.

Next, the statistically significant LR test implies that health status and physical activity are endogenous in the equation of non-prescribed medicines, overnight and day hospitalization, visits to generalist practitioner, and home healthcare services. Therefore, in order to tackle the endogeneity of both physical activity and health status, the three instrumental variables mentioned above are introduced. These results are presented in full in Tables (A12)–(A19) from Appendix. Both parents’ education and individuals’ attitude concerning the future have the expected signs and are jointly significant in the health status equation. In other words, respondents’ whose parents did go to school and those not being preoccupied about their future have a higher probability to be in good health, while those being more pessimistic about their future have a higher probability to be in poor health. Moreover, the instrumental variable in physical activity equation is also significantly associated with physical activity, suggesting that individuals are more likely to be more physically active if they are members of an association or sport club. Further, performing the overidentification test, the Chi-square values for the LR test indicate that the null hypothesis cannot be rejected, therefore healthcare utilization is not directly linked to none of the three instrumental variables. Thus, their effects are reflected to some extent on the relationship between healthcare utilization and the two endogenous variables, i.e. health status and physical activity.

In this regard, comparing the probit estimates to recursive multivariate probit estimates shows that these are not different in sign, but are larger after controlling for endogeneity. Both moderately and highly active people are statistically different from their low active counterparts in being higher users of generalist physician’s services. Being moderate and high physically active is also associated with a lower probability to be hospitalized overnight or to use home healthcare services over the 12-month study period. Interestingly, after considering the reverse causality between physical activity, individuals’ health status and the use of non-prescribed medicines, the difference between moderate and low active people becomes statistically significant. In contrast, moderately and highly active individuals were as likely as their low active counterparts to consume prescribed medicines, to be hospitalized during the day, or to be high users of specialist services.

Lastly, it is noteworthy that better health status and decrease in age are strongly associated with lower use of each healthcare service. Besides these two important determinants, our results highlight that women are higher consumers of non-prescribed and prescribed medicines and are more likely to be hospitalized overnight, to use generalist and specialist physician services, preventive services, or home healthcare compared to their male counterparts. In this age group, significant and strong associations are found as well between education and non-prescribed medicines consumption, overnight hospitalization, generalist and specialist practitioner services. The results on the relationship between education and healthcare utilization reveal that respondents with higher levels of education are more likely to use these healthcare services than those having a low level of education. Finally, the findings suggest that health behaviour factors are associated to a lesser extent to healthcare utilization.

#### ≥ 65 years age group

The results of the regression analyses pertaining to the 65 years and older age group are presented in the last two columns in Table 2. In this regard, without accounting for potential endogeneity, statistically significant associations were found only between physical activity and the use of non-prescribed medicines, overnight hospitalization, and home healthcare services. Within this age group, moderately active individuals were less likely to need home healthcare services, but as likely as their low active counterparts to consume non-prescribed drugs and to be overnight hospitalized. As for the respondents pertaining to the highest level of physical activity, the results suggest that they were significantly more likely to consume non-prescribed medicines and less likely to use home healthcare services or to be hospitalized compared to those reporting a low level of physical activity.

As before, comparison of the probit and recursive multivariate probit estimates shows that the estimates on the relationship between physical activity and healthcare services are larger and strongly significant when controlling for endogeneity, but only in relation to visits to generalist physician and home healthcare services. Dealing with the reverse causality of both physical activity and health status index reveals larger and statistically significant associations between generalist physician services and physical activity. While the probability of using generalist practitioner services increases with the intensity of physical activity, we also found that female respondents are lower users of such services, as well as older adults with lower level of education, lower level of income, without insurance, or as those who are smokers (as shown in Table A16). In contrast, negative association was found between the use of home healthcare and physical activity. Similarly, married individuals, as well as tobacco and alcohol consumers were less likely to use home healthcare services, while those aged 75 years and older, as well as obese older adults were more in need of this particular healthcare (as shown in Table A19).

Finally, as for the other samples, among the 65 years and older age group, the most substantial association is between the outcome variables and health status, indicating that better health is significantly associated with lower use of any of healthcare services. Therefore, due to their more fragile health status, the link between physical activity and healthcare utilization is less obvious among respondents aged 65 years and older compared to those aged under 65 years. Within older adults group, significant differences in the use of healthcare services are found especially between individuals aged 65–70 years and those aged 75 years and older.

### Other robustness checks

To investigate the robustness of our results, we further conduct an additional analysis by considering the age of 60 as the grouping limit of the sample. This analysis will reveal if the relation between physical activity and healthcare utilization suffers major modifications in sign, magnitude and significance of the coefficients of interest. The results of the regression analyses pertaining to both years age groups are presented in Table 3.

**Table 3:** The association between physical activity and healthcare utilization, stratified by age

	***<***60 Years Age Group				***≥*** 60 Years Age Group			
	Probit Model		Recursive Multivariate Probit Model		Probit Model		Recursive Multivariate Probit Model	
**Non-prescribed medicines**								
Moderately active	0.0554	*p* < .1	0.1050	*p* < .001	0.1152	*p* < .1	0.0909	*p* < .1
Highly active	0.0858	*p* < .1	0.1465	*p* < .001	0.2115	*p* < .001	0.1626	*p* < .1
*Test of endogeneity*	–		5.52	*p* < .01	–		0.10	*p* < .1
**Prescribed medicines**								
Moderately active	−0.0410	**.**	− 0.0405	*p* < .1	− 0.0599	*p* < .1	−0.1134	*p* < .1
Highly active	−0.1016	*p* < .01	−0.1129	*p* < .01	−0.1298	*p* < .1	−0.2016	*p* < .1
*Test of endogeneity*	–		0.01	*p* < .1	–		0.25	*p* < .1
**Overnight hospitalization**								
Moderately active	−0.1221	*p* < .01	−0.1440	*p* < .001	−0.1119	*p* < .1	−0.1139	*p* < .1
Highly active	−0.0785	*p* < .1	−0.1151	*p* < .01	−0.1588	*p* < .01	−0.1473	*p* < .1
*Test of endogeneity*	–		4.21	*p* < .01	–		0.19	*p* < .1
**Day hospitalization**								
Moderately active	−0.0083	*p* < .1	−0.0726	*p* < .01	0.0517	*p* < .1	0.1093	
Highly active	−0.0682	*p* < .1	−0.1136	*p* < .001	0.0591	*p* < .1	0.1038	
*Test of endogeneity*	–		3.87	*p* < .01	–		0.73	*p* < .1
**Generalist physician services**								
Moderately active	0.2088	*p* < .001	0.2286	*p* < .001	0.1731	*p* < .1	0.2615	*p* < .01
Highly active	0.2337	*p* < .001	0.2876	*p* < .001	0.0817	*p* < .1	0.2613	*p* < .01
*Test of endogeneity*	–		4.37	*p* < .01	–		6.16	*p* < .01
**Specialist physician services**								
Moderately active	0.0467	*p* < .1	0.0059	*p* < .1	0.0129	*p* < .1	0.0576	*p* < .1
Highly active	0.0532	*p* < .1	0.0049	*p* < .1	0.0147	*p* < .1	0.1455	*p* < .1
*Test of endogeneity*	–		4.84	*p* < .01	–		2.29	*p* < .1
**Preventive services**								
Moderately active	0.0987	*p* < .01	0.0421	*p* < .1	−0.0170	*p* < .1	− 0.0255	*p* < .1
Highly active	0.0881	*p* < .01	0.0218	*p* < .1	−0.0385	*p* < .1	−0.0512	*p* < .1
*Test of endogeneity*	–		1.40	*p* < .1	–		0.55	*p* < .1
**Home healthcare services**								
Moderately active	−0.1690	*p* < .001	−0.1601	*p* < .001	− 0.2556	*p* < .001	−0.3155	*p* < .001
Highly active	−0.1895	*p* < .001	−0.2022	*p* < .001	−0.3432	*p* < .001	−0.4596	*p* < .001
*Test of endogeneity*	–		13.78	*p* < .001	–		5.11	*p* < .01

(1) The table reports estimates for two approaches (probit and recursive multivariate probit) on the association between physical activity and the use of medicines and healthcare services. The coefficients indicate the difference between the probit index (Z-score) of each category of physical activity, i.e. moderately and highly physically active, and that corresponding to the reference group for physical activity, which is low physically active. (2) The relationship between physical activity and healthcare utilization is controlled by the following variables: sex of respondent; age group; education level; legal marital status; employment status; insurance; income level; BMI status; smoking; alcohol consumption risk profile; nutrition – fruits and vegetables consumption; health status index

*Source:* Authors’ computation

For people aged under 60 years, the modelling results are similar to those obtained for the under 65 age group. The only notable difference is related to the association (regardless of its intensity) between physical activity and healthcare utilization (excepting generalist practitioner visits), which is slightly larger in the latter age group. As for the groups of older adults, the main difference refers to the relationship between physical activity and overnight hospitalization, which among adults age 60 years and older, the association between physical activity and these particular healthcare services is slightly stronger for highly active group in comparison to 65 years and older age group.

## Discussion

The modelling results pertaining to the entire population showed that higher physical activity was associated with lower prescribed medicines consumption, lower use of overnight hospitalization, specialist visits, and home healthcare services, but with higher use of generalist practitioner and preventive services. However, the associations with non-prescribed medicines consumption and day hospitalization were not statistically significant, as well as the difference between moderately active group and low active one in using specialist services. Overall, our findings align well with the literature, suggesting that being moderately and highly physically active is associated with a lower probability of using prescription medication [37, 58, 61], being hospitalized overnight [37, 59, 60], or using home healthcare services over the 12-months study period [37, 59]. In contrast, our analysis of the use of some healthcare services in relation to physical activity provides contradictory results compared to other studies. Even if there is indeed an obvious deviation from the expected overall conclusion based on the related line of research, our outcomes still find their support in few studies. In compliance with Maresova and Vokoun [60] and Kang and Xiang [37], individuals reporting to be more physically active were more likely to have a higher use of generalist practitioner and preventing services compared to low active people. In addition, moderately and highly active people were no more or less likely to use non-prescribed medicines, to be hospitalized during the day [37], or to be higher users of specialist physician services [37, 60] than their low active counterparts. These results suggest that the associations between physical activity and healthcare utilization should be analyzed in relation to the specificities of the population under study and also considering the other relevant socioeconomic, demographic, and health behaviour determinants, both of which could provide important insights into this link.

Among the respondents under 65 years, the relationship between physical activity and the use of healthcare depends on the type of healthcare service: significantly positive association between physical activity and non-prescribed drugs consumption, preventive services, and visits to general practitioner; lower consumption of prescribed medicines and lower use of inpatient and home healthcare services with higher physical activity; no significant association between physical activity and specialist physician visits. These results are to some extent comparable with findings from previous studies estimating the association of physical activity with healthcare utilization in adults’ population. For instance, Kang and Xiang [37] showed that adults who engaged in physical activity were more likely to use preventive and office-based services and were less likely to use inpatient care, home healthcare services, and prescription medicines. Fisher et al. [39] found generalist physician visits to be inversely associated with physical activity in the 50 to 64 years age group, but no significant association between physical activity and specialist services or overnight hospitalization. Mitchell et al. [107] also showed a strong negative relationship between physical activity and physician services among men aged 20–79 years. Similarly, Denkinger et al. [38] found physician visits to be inversely associated with physical activity in older adults, but no relationship with overnight or day hospitalization. Bertoldi et al. [58], as well as Denkinger et al. [38], showed that the level of physical activity is inversely associated with the prevalence and number of medicines used in a population-based sample of adults. However, as Fisher et al. [39] state, the findings in this area are somewhat equivocal, which is due, in part, to considerable variation in sample populations, study design, and methods.

Within the older adults group, the results reveal fewer significant associations between physical activity and the use of different healthcare services. In this regard, significant differences in using some of these healthcare services are found especially between highly active older adults and their low active counterparts. Therefore, lower use of inpatient (overnight hospitalization) and home healthcare services are related to higher physical activity, while positive associations between non-prescribed medicines consumption and generalist visits and physical activity are found. Our study findings are partially consistent with reports from several prior studies. For instance, the results of Wang et al. [56] indicated that over-65 individuals engaged in regular physical activities were less likely to use outpatient services, ER visits, or to be hospitalized. Similarly, Nguyen et al. [42] showed that older individuals suffering from diabetes who participated in a community-based exercise program had fewer hospitalizations than those enrollers who did not attend the program. Woolcott et al. [46] and Sari [43] found as well that physical activity was associated with a decreased likelihood of hospitalization among Canadians aged 65 years and older. Examining the association between physical activity and unplanned hospital admissions in a diverse cohort of UK city-dwelling older people aged 70 and over, Simmonds et al. [45] concluded that low active individuals was almost twice as likely to be vulnerable than the high active ones. The same authors found a similar strong relationship between physical activity and medical prescriptions, suggesting that both low and moderate active groups received approximately 50% more prescriptions than the high group. This supports the findings of Silva et al. [44], who showed that higher levels of physical activity were significantly associated with lower use of medication in elderly women engaged in a physical-activity program. In the study of Denkinger et al. [38], reduced physical activity was also among the best predictors of both medicines consumption and physician contacts in community dwelling older adults aged 65–90 years. In contrast, the findings of Fisher et al. [39] revealed that active respondents were actually more likely to report more nights in hospital. The authors’ explained that these differences in healthcare utilization are due to considerable heterogeneity within the older adult population relative to physical activity, health status, and healthcare services. Thereby, working with a stratification of the sample into more groups for older adults may accurately assess the association between the use of different types of healthcare services and physical activity. Overall, the results support that it is essential for older adults too to get involved in physical activities.

Even though the data used come from a 2014 national survey among the French population, our study presents a range of empirical evidence showing the importance of physical activity for the use of health service. A higher level of physical activity for each age group may contribute to better health status of the population and therefore substantially reduce costs to the health system. These results can be correlated with those showing that in France, despite the programs implemented to increase physical activity and awareness of its importance for population health, physical activity levels did not increase significantly between 2006 and 2016 [82, 83]. In this respect, in order to increase physical activity as a health prevention tool, various programs are already implemented in France. Among these policies, the more recent are the legislative decree approved in 2016 allowing health professionals to prescribe physical activities adapted to patients with long-term medical conditions, and the adoption of a care package including a medical-sports evaluation for patients with cancer. Moreover, the Ministry of Sport and the Ministry of Solidarity and Health set up “Maisons Sport-Santé” as part of the National Strategy for Sport and Health 2019–2024. The aim of this program is to encourage as many people as possible to integrate physical activity and sport into their daily lives in a regular, sustainable and adapted way in order to improve the health of the population. Our results show that current and future policies should target populations most at risk and therefore envision actions that public decision-makers or insurers could specifically fund. For instance, an effort should be made to promote cycling and walking in public spaces. This implies a real strategy for urban spatial planning with more cycle lanes and pedestrian areas (in this respect, France is far behind Germany and the Netherlands). The introduction of more sports in schools should also be developed; this involves a review of school curricula by the Ministry of Education. The number of sports and leisure facilities in France could also be increased to create spaces for physical activity (parks in towns, swimming pools, gyms, etc.). In conclusion, it is no longer necessary to think of different policies “in silos”, but rather to have an “integrated” policy combining different policies in different areas (town planning, health, education, urban policy, etc.).

The above-mentioned remarks are also strengthened by the well-known fact that regular, progressive and supervised physical and sports activities can prevent 30% of cardiovascular diseases, 20 to 25% of breast or colon cancers, 50% of type 2 diabetes and 30% of strokes. It also reduces the risk of Alzheimer’s disease and delays the onset of loss of autonomy by 7 to 10 years. The regular practice of physical activities also makes possible the increase of healthy life expectancy, an indicator which has not changed significantly in France for more than 10 years and which remains below the European average. Therefore, promoting and increasing physical and sports activities could contribute to major societal issues, knowing that in France there are 10 million people with long-term medical conditions and 20 million people suffering from chronic diseases, and that the cost of sedentary and chronic diseases is estimated at 17 billion Euros per year.

### Strengths and limitations

According to the existing literature and to the results obtained, the strengths of our study can be summarized in at least three points. Firstly, it provides an overall analysis of the context of healthcare utilization in relation to physical activity in a nationally (French) representative sample. To the best of our knowledge, this study is among a small few to examine the association between physical activity and different types of healthcare utilization using the EHIS-ESPS 2014 survey data. Secondly, our study relies on the assessment of a general physical activity index which includes the participation frequency and activity duration in walking, riding a bike, making sports, and muscle building exercises. Within this context, another notable difference between this analysis and previous studies was the inclusion of age into the index measurement methodology in order to obtain a more accurate classification of the population depending on the type and intensity of physical activity. Thirdly, the methodological approach employed in the empirical analysis enables to cope with the problem of endogeneity caused by unobserved heterogeneity and possible reverse causality of healthcare utilization in relation to both physical activity and health status. Moreover, the association between healthcare utilization and physical activity was explored both for the whole population (classifying all respondents as one homogeneous group) and separately for two age groups (15 to 64 years and 65 years and older). The sample stratification enabled to account for variations in health between age groups that may affect both the level of physical activity and healthcare utilization.

Our analysis is also marred by limitations. Firstly, the self-reported nature of data, related especially to physical activity, health status and healthcare utilization, leads to the possibility of response bias due to inaccurate recall or social desirability. In this regard, we replaced self-reported health with a composite health status index based on the SF-36 scoring methodology, but adapted to a large extent to the available data. Furthermore, the cross-sectional nature of the data limits the exploitation of reverse causality between healthcare utilization and some of its determinants. In this case, controlling for the potential endogeneity of both physical activity and health status would be a difficult task as it involves the use of instrumental variables estimation. Therefore, another limitation of our study is related in fact to the choice of these variables that depends again on the used survey data. Although we were able to address to some extent the endogeneity issue using these instruments, our results should be interpreted with caution, as longitudinal effects of physical activity on healthcare utilization could not be captured. This aspect is even more highlighted by the reverse causality between physical activity and the use of different health services. In other words, based on cross-sectional data, the impact over time of physical activity on health cannot be captured, nor can the effect of using certain health services on physical activity be assessed. For the latter observation, as Sari [59] mentions, a person may be physically inactive for a certain period of time because of health problems, which may require medical interventions that prevent her or him from engaging in physical activities.

Considering these limitations, a future research direction could be a comparative study on the prevalence of physical activity in France in 2014 and 2020, based on the third wave of the EHIS survey. Such a research could highlight whether policies on physical activity development in France in recent years have had the desired effect. Finally, it could suggest the development of new tools to encourage the population to become increasingly involved in physical activity and sport. Even if panel data are not available, the replication of the empirical analysis on the relationship between physical activity and healthcare utilization and the data from the third wave of the survey could provide new evidence on the role of physical activity in reducing health service costs.

## Conclusions

Overall, our empirical evidences underline that the relation between healthcare utilization and physical activity in French population depends on the type of healthcare services and the age group. In this respect, we observe a statistical significant relationship between physical activity and prescribed medicines consumption, day hospitalization and preventive services use only among adult respondents. Common to both age groups, the positive association between physical activity and the use of general physician services and non-prescribed medicines consumption reveal that moderately and highly active adults and elders may be more health conscious and therefore may seek referrals to generalist and other prevention measure more frequently than their inactive counterparts. This possible explanation could be sustained also by the negative response of the use of inpatient (especially overnight hospitalization) and home healthcare services to higher physical activity.

Consequently, our findings highlight the double role of physical activity on health, that of preventive measure and of treatment prescribed as part of the care of patients with different medical conditions. Moreover, this study provides a significant contribution to a growing body of evidence suggesting significant strong associations between physical activity and healthcare utilization at the level of French population. Interventions aimed at increasing physical activity may result in significant reductions in the demand of healthcare services, and indirectly in lowering the public health related costs. Thus, our paper gives important insights for policy-makers about the potential impact of population-based strategies to increase physical activity participation among French people on healthcare utilization. In addition, considering the stratification of population by age, the recommendations on physical activity for health should be addressed separately depending on the specificity and the most relevant determinants of healthcare utilization corresponding to each age group.

## Supplementary Information

**Additional file 1: Appendix.** Tables A1-A19b.

## Data Availability

This paper is based on data from Eurostat, European Health Interview Survey (EHIS) 2014. We obtained these data by applying a research project proposal at institutional level, according to the criteria required by Eurostat, as mentioned on the official webpage: https://ec.europa.eu/eurostat/web/microdata/european-health-interview-survey. The EHIS 2014 data are official, public, and institutionally available from Eurostat. The responsibility for all conclusions drawn from the data lies entirely with the authors.
