# Supplementary material for: Physical activity and healthcare utilization in France: evidence from the European Health Interview Survey (EHIS) 2014
Source: BMC Public Health. 2022 Jul 15;22:1355. doi: 10.1186/s12889-022-13479-0 (PMC9288017; doi:10.1186/s12889-022-13479-0)
Supplement: Supplementary file 1 — Additional file 1: Appendix. Tables A1-A19b. [file 12889_2022_13479_MOESM1_ESM.pdf]

Appendix

Table A1 Data used

	Class of indicators			Indicators	Measurements
**Healthcare utilization**	medicine use			self-medications	use *versus* non-use
				prescribed medications	use *versus* non-use
	healthcare services utilization	inpatient services	hospital services	nights spent in a hospital	yes *versus* no
				days spent in a hospital	yes *versus* no
			home healthcare	home visits from health professional	yes *versus* no
		outpatient services	generalist	visits to physician	yes *versus* no
			specialist	visits to physician	yes *versus* no
		preventive services		dental check-up	yes *versus* no
				flu shot	yes *versus* no
				blood pressure check-up	yes *versus* no
				cholesterol check-up	yes *versus* no
				blood glucose test	yes *versus* no
				immunological test	yes *versus* no
				general index	cautious (using at least 2 preventive measures annually); incautious (otherwise)
**Physical activity**	transport	walking (1)		number of days per week	
		walking (2)		duration per day	< 10 minutes per day<br>10 - 29 minutes per day<br>30 - 59 minutes per day<br>1 - 2 hours per day<br>2 - 3 hours per day<br>>= 3 hours per day
		riding a bike (1)		number of days per week	
		riding a bike (2)		duration per day	< 10 minutes per day<br>10 - 29 minutes per day<br>30 - 59 minutes per day<br>1 - 2 hours per day<br>2 - 3 hours per day<br>>= 3 hours per day
	leisure	making sports (1)		number of days per week	
		making sports (2)		duration per day	< 10 minutes per day<br>10 - 29 minutes per day<br>30 - 59 minutes per day<br>1 - 2 hours per day<br>2 - 3 hours per day<br>>= 3 hours per day
		muscle building exercises		number of days per week	
**Control variables**	individual characteristics			age	15 years and over
				gender	male; female
				education level	low; middle; high; student
				marital status	married; unmarried (single, concubine, widower, divorced)
				employment status	employed; unemployed (respondents in school or not attending school, and without job)
				income level	< 1500 euros; > 1500 euros
				health insurance	without; private; complementary insurance
	health status characteristics			health status index	poor health; fair health; moderate health; good health
	health behaviours			Body Mass Index	normal weight (< 25 kg/m^2);<br>overweight (25-29.9 kg/m^2);

			obese (≥ 30 kg/m^2)
		smoking status	non-smoker; occasional smoker; light or heavy daily smoker
		drinking profile	no-risk (non-consumer, safe consumer); risky (one-time risk consumer, chronic and dependent consumer)
		nutrition (frequency of eating fruits and vegetables and its quantity on a daily basis)	insufficient (do not respect the recommendations of eating at least 5 fruits and vegetables on a daily basis); sufficient (otherwise)

Descriptive statistics

Table A2 Control variables, both for the entire population and stratified by age group and physical activity level

Control variables	Total (%)			< 65 years (%)			≥ 65 years (%)		
	Physical activity			Physical activity			Physical activity		
	Low	Moderate	High	Low	Moderate	High	Low	Moderate	High
Age (15-24)	1.61	0.89	9.31	2.24	1.11	12.06	-	-	-
Age (25-44)	34.48	40.40	36.74	48.03	50.49	47.36	-	-	-
Age (45-64)	35.71	38.73	31.16	49.74	48.40	47.58	-	-	-
Age (65-74)	12.00	12.00	14.61	-	-	-	42.52	60.06	64.13
Age (75-84)	11.11	6.71	7.26	-	-	-	39.40	33.57	31.86
Age (≥85)	5.10	1.27	0.91	-	-	-	18.08	6.37	4.01
Sexe (Male)	46.13	43.42	54.66	48.11	42.79	54.31	41.07	45.96	55.86
Sexe (Female)	53.87	56.58	45.34	51.89	57.21	45.69	58.93	54.04	44.14
Education (High)	6.65	16.62	14.81	7.52	18.44	15.13	4.43	9.35	13.70
Education (Middle)	21.36	26.99	24.97	26.11	29.94	26.55	9.20	15.25	19.61
Education (Low)	68.57	50.07	44.94	61.61	43.72	38.53	86.36	75.40	66.69
Education (Student)	3.42	6.32	15.29	4.76	7.90	19.79	-	-	-
Marital_Status (Married)	59.98	60.37	49.91	56.49	57.42	42.49	68.86	72.15	75.04
Marital_Status (Unmarried)	40.02	39.63	50.09	43.51	42.58	57.51	31.14	27.85	24.96
Employment (Employed)	47.78	51.56	40.66	65.96	64.10	52.33	1.45	1.36	1.12
Employment (Unemployed)	52.22	48.44	59.34	34.04	35.90	47.67	98.55	98.64	98.88
Insurance (CMU-C)	8.46	8.08	8.16	11.15	9.78	10.35	1.60	1.37	0.81
Insurance (No_CS)	4.78	3.45	4.19	4.59	3.39	4.50	5.25	3.68	3.16
Insurance (Private_CS)	86.76	88.47	87.65	84.26	86.84	85.15	93.15	94.95	96.04
Income (<1500)	59.47	47.12	45.84	58.00	47.18	47.11	63.41	46.86	41.41
Income (>1500)	40.53	52.88	54.16	42.00	52.82	52.89	36.59	53.14	58.59
BMI_Status (Normal weight)	44.13	51.88	61.34	48.18	55.43	65.80	33.72	37.58	46.39
BMI_Status (Obese)	23.36	15.60	10.29	20.19	14.35	9.41	31.50	20.66	13.24
BMI_Status (Overweight)	32.51	32.52	28.37	31.63	30.22	24.79	34.78	41.76	40.37
Smoking (Daily)	27.82	20.52	18.36	35.83	23.74	21.51	6.05	6.69	7.13
Smoking (Occasional)	4.05	5.74	6.46	4.96	6.58	7.50	1.60	2.12	2.78
Smoking (Never)	68.13	73.73	75.18	59.21	69.68	70.99	92.35	91.19	90.09
Drinking (Risky)	24.43	28.31	29.54	29.21	31.50	32.85	12.28	15.53	18.30
Drinking (Not risky)	75.57	71.69	70.46	70.79	68.50	67.15	87.72	84.47	81.70
Nutrition (Insufficient)	26.75	17.61	17.33	31.54	19.74	20.19	14.48	9.00	7.53
Nutrition (Sufficient)	73.25	82.39	82.67	68.46	80.26	79.81	85.52	91.00	92.47
Health_Index (Good health)	21.44	26.86	30.72	26.70	30.08	34.52	7.91	13.84	17.81
Health_Index (Moderate)	17.86	24.53	24.24	21.43	25.97	24.66	8.70	18.75	22.80
Health_Index (Fair health)	24.13	25.59	24.88	25.21	25.21	23.88	21.36	27.33	28.28
Health_Index (Poor health)	36.57	23.02	20.16	26.66	18.78	16.94	62.03	40.09	31.00

Assessment of physical activity levels

Table A3 Active Transportation Domain

Continuous scores	Active Transportation Domain
walking MET-minutes/ week for transport	**W** = **3.3** * walking minutes * walking days for transportation
cycle MET-minutes/ week for transport	**C** = **6.0** * cycling minutes * cycling days for transportation
total transport MET-minutes/week	**Total PA** = Walking + Cycle MET-minutes/week scores for transportation

Table A4 Leisure-Time Domain

Continuous scores	Leisure-Time Domain
walking MET-minutes/ week leisure	**W** = **3.3** * walking minutes * walking days in leisure
moderate MET-minutes/ week leisure	**M** = **4.0** * moderate-intensity activity minutes * moderate days in leisure
vigorous-intensity MET-minutes/ week leisure	**V** = **8.0** * vigorous-intensity activity minutes * vigorous-intensity days in leisure
total leisure-time MET-minutes/week	**Total PA** = Walking + Moderate + Vigorous MET-minutes/week scores in leisure

Table A5 WHO's recommendations for physical activity depending on age

Type / Age group	Cardiorespiratory	Muscle building	Physical activity for balance
**15-17 years**	less than 60 min per day of physical activities of moderate to high intensity **or** at least 15 min per day of physical activities of high intensity, at least 3 days per week	3 sessions of 20 minutes per week	-
**18-65 years**	less than 30 min per day of physical activities of moderate to high intensity, at least 5 days per week	1 to 2 times per week, with 1 to 2 days of recovery between two sessions	-
**> 65 years**	less than 30 min per day of moderate physical activity **or** less than 15 min per day of intense physical activity **or** a combination of moderate and intensive physical activities, at least 5 times per week	2 or more than 2 days per week	less than 2 times per week

Table A6 MET's degree of intensity

Degree of intensity	MET
Sedentary activities	< 1,6 MET
Activities of low intensity	[1,6 – 3) MET
Activities of moderate intensity	[3 – 6) MET
Activities of high intensity	[6 – 9) MET
Activities of very high intensity	> = 9 MET

Table A7 Adjusted physical activity levels by age group

Group	15 – 17 years	18 – 65 years	> 65 years
**low physically active**	< 3 ds/wk and < 1 h/d of *walking* **or** < 3 ds/wk and < 10 min/d of *riding a bike* **or** *making sports* **or** < 3 ds/wk of *muscle building exercises*	< 5 ds/wk and < 1 h/d of *walking* **or** < 5 ds/wk and < 30 min/d of *riding a bike* **or** *making sports* **or** 0 ds/wk of *muscle building exercises*	< 5 ds/wk and < 30 min/d of *walking* **or** < 5 ds/wk and < 10 min/d of *riding a bike* **or** *making sports* **or** < 2 ds/wk of *muscle building exercises*
**moderate physically active**	≥ 3 ds/wk and 1-2 h/d of *walking* **or** ≥ 3 ds/wk and 10 min-2 h/d of *riding a bike* **or** *making sports* **or** ≥ 3 ds/wk of *muscle building exercises*	≥ 5 ds/wk and 1-2 h/d of *walking* **or** ≥ 5 ds/wk and 30 min-2 h/d of *riding a bike* **or** *making sports* **or** ≥ 1 ds/wk of *muscle building exercises*	≥ 5 ds/wk and 30 min-2 h/d of *walking* **or** ≥ 5 ds/wk and 10 min-2 h/d of *riding a bike* **or** *making sports* **or** ≥ 2 ds/wk of *muscle building exercises*
**high physically active**	≥ 3 ds/wk and ≥ 2 h/d of *walking*, **or** ≥ 3 ds/wk and ≥ 2 h/d of *riding a bike* **or** *making sports* **or** ≥ 3 ds/wk of *muscle building exercises*	≥ 5 ds/wk and ≥ 2 h/d of *walking*, **or** ≥ 5 ds/wk and ≥ 2 h/d of *riding a bike* **or** *making sports* **or** ≥ 1 ds/wk of *muscle building exercises*	≥ 5 ds/wk and ≥ 2 h/d of *walking*, **or** ≥ 2 ds/wk and ≥ 2 h/d of *riding a bike* **or** *making sports* **or** ≥ 2 ds/wk of *muscle building exercises*

Notes: wk stands for weeks; *ds* stands for days; *h* stands for hours; *min* stands for minutes

Modelling results

Table A8 Non-prescribed medicines

Variables	Non-prescribed medicines		Health Index		Physical activity	
Intercept	-1.3566	p<.001	0.9874	p<.001	-0.4691	p<.001
PA (Moderate)	0.1049	p<.001	0.1643	p<.001	-	
PA (High)	0.1540	p<.001	0.2433	p<.001	-	
Age (25-44)	0.1279	p<.1	0.0053	p<.1	0.8841	p<.001
Age (45-64)	0.1867	p<.01	0.2582	p<.001	0.8713	p<.001
Age (65-74)	0.2399	p<.01	0.4381	p<.001	0.7722	p<.001
Age (75-84)	0.2323	p<.01	0.9063	p<.001	1.0276	p<.001
Age (≥85)	0.2864	p<.01	1.3236	p<.001	1.6278	p<.001
Sexe (Female)	0.2586	p<.001	0.2895	p<.001	0.2020	p<.001
Education (High)	0.4042	p<.001	-0.1152	p<.001	-0.1420	p<.001
Education (Middle)	0.2365	p<.001	-0.0746	p<.001	-0.1089	p<.001
Education (Student)	0.2398	p<.001	-0.3362	p<.001	-0.1621	p<.001
Marital_Status (Married)	0.0134	p<.1	-0.0588	p<.01	0.1492	p<.001
Employment (Employed)	0.0856	p<.01	-0.1711	p<.001	0.2247	p<.001
Insurance (CMU-C)	-0.1696	p<.001	0.1728	p<.001	-0.1431	p<.001
Insurance (No_CS)	0.0732	p<.1	-0.0023	p<.1	-0.0596	p<.1
Income (<1500)	-0.0818	p<.001	0.1379	p<.001	0.1137	p<.001
BMI_Status (Normal_weight)	0.1028	p<.001	-0.0832	p<.001	-0.1601	p<.001
BMI_Status (Obese)	-0.1042	p<.01	0.2070	p<.001	0.2106	p<.001
Smoking (Daily)	-0.0472	p<.1	0.1025	p<.001	0.1841	p<.001
Smoking (Occasional)	0.1037	p<.01	0.1151	p<.01	-0.0419	p<.1
Drinking (Risky)	0.1224	p<.001	0.0875	p<.001	-0.0395	p<.1
Nutrition (Insufficient)	-0.2084	p<.001	0.0565	p<.01	0.1878	p<.001
Health_Index (Good health)	-0.6607	p<.001	-		0.2457	p<.001
Health_Index (Moderate health)	-0.2412	p<.001	-		0.2570	p<.001
Health_Index (Fair health)	-0.1745	p<.001	-		0.1768	p<.001
Parents_Education (Non-response)	-		-0.3686	p<.001	-	
Parents_Education (With school)	-		-0.4640	p<.001	-	
Attitude_Future (Not preoccupied)	-		0.0778	p<.001	-	
Attitude_Future (Non-response)	-		0.2547	p<.001	-	
Member_Association (No)	-		-		-0.2850	p<.001

Notes: (1) The coefficients indicate the difference between the probit index (Z-score) of each category of each independent variable and that corresponding to the reference category of each independent variable. (2) The reference categories for the independent variables are: *low active* (physical activity); *15-24 years* (age group); *male* (sex of respondent); *primary education level* (education); *unmarried* (legal marital status); *unemployed* (employment status); *private health insurance* (insurance); *more than 1500 euros* (income level); *overweight* (BMI status); *never* (smoking); *no-risk* (alcohol consumption risk profile); *recommended* (nutrition – fruits and vegetables consumption); *poor health* (health index). (3) The reference categories for each instrumental variable are: *without school* (parents' education); *preoccupied* (attitude towards future); *yes* (membership in an association).

Table A9 Generalist physician services

Variables	Generalist services		Health Index		Physical activity	
Intercept	1.4545	p<.001	0.9786	p<.001	-0.5017	p<.001
PA (Moderate)	0.2090	p<.001	0.1638	p<.001	-	
PA (High)	0.2106	p<.001	0.2377	p<.001	-	
Age (25-44)	-0.0517	p<.1	0.0131	p<.1	0.9049	p<.001
Age (45-64)	-0.0438	p<.1	0.2619	p<.001	0.8923	p<.001
Age (65-74)	0.2234	p<.05	0.4394	p<.001	0.7876	p<.001
Age (75-84)	0.4558	p<.001	0.9033	p<.001	1.0541	p<.001
Age (≥85)	0.4727	p<.01	1.3381	p<.001	1.6510	p<.001
Sexe (Female)	0.2721	p<.001	0.2894	p<.001	0.2042	p<.001
Education (High)	-0.1433	p<.001	-0.1201	p<.001	-0.1376	p<.001
Education (Middle)	-0.0308	p<.1	-0.0805	p<.001	-0.1078	p<.001
Education (Student)	0.1622	p<.01	-0.3462	p<.001	-0.1577	p<.001
Marital_Status (Married)	0.1176	p<.001	-0.0600	p<.01	0.1507	p<.001
Employment (Employed)	-0.0473	p<.1	-0.1755	p<.001	0.2234	p<.001
Insurance (CMU-C)	0.0072	p<.1	0.1732	p<.001	-0.1375	p<.001
Insurance (No_CS)	-0.5544	p<.001	0.0016	p<.1	-0.0563	p<.1
Income (<1500)	-0.0848	p<.01	0.1420	p<.001	0.1124	p<.001
BMI_Status (Normal_weight)	-0.0692	p<.05	-0.0852	p<.001	-0.1572	p<.001
BMI_Status (Obese)	0.1447	p<.001	0.2053	p<.001	0.2123	p<.001
Smoking (Daily)	-0.1828	p<.001	0.0985	p<.001	0.1905	p<.001
Smoking (Occasional)	-0.0497	p<.1	0.1078	p<.01	-0.0360	p<.1
Drinking (Risky)	-0.0748	p<.001	0.0916	p<.001	-0.0397	p<.1
Nutrition (Insufficient)	-0.0650	p<.01	0.0568	p<.01	0.1872	p<.001
Health_Index (Good health)	-0.6960	p<.001	-		0.2364	p<.001
Health_Index (Moderate health)	-0.4756	p<.001	-		0.2525	p<.001
Health_Index (Fair health)	-0.2906	p<.001	-		0.1697	p<.001
Parents_Education (Non-response)	-		-0.3626	p<.001	-	
Parents_Education (With school)	-		-0.4502	p<.001	-	
Attitude_Future (Not preoccupied)	-		0.0868	p<.001	-	
Attitude_Future (Non-response)	-		0.3056	p<.001	-	
Member_Association (No)	-		-		-0.2877	p<.001

Notes: (1) The coefficients indicate the difference between the probit index (Z-score) of each category of each independent variable and that corresponding to the reference category of each independent variable. (2) The reference categories for the independent variables are: *low active* (physical activity); *15-24 years* (age group for those younger than 65 years old); *65-69 years* (age group for those of 65 years and older); *male* (sex of respondent); *primary education level* (education); *unmarried* (legal marital status); *unemployed* (employment status); *private health insurance* (insurance); *more than 1500 euros* (income level); *overweight* (BMI status); *never* (smoking); *no-risk* (alcohol consumption risk profile); *recommended* (nutrition – fruits and vegetables consumption); *poor health* (health index). (3) The reference categories for each instrumental variable are: *without school* (parents' education); *preoccupied* (attitude towards future); *yes* (membership in an association).

Table A10 Home healthcare services

Variables	Non-prescribed medicines		Health Index		Physical activity	
Intercept	-0.6533	p<.001	0.9513	p<.001	-0.5056	p<.001
PA (Moderate)	-0.2152	p<.001	0.2485	p<.001	-	
PA (High)	-0.2599	p<.001	0.1709	p<.001	-	
Age (25-44)	0.1188	p<.1	0.0093	p<.1	0.9035	p<.001
Age (45-64)	0.1657	p<.1	0.2649	p<.001	0.8948	p<.001
Age (65-74)	0.3790	p<.001	0.4404	p<.001	0.7992	p<.001
Age (75-84)	0.5725	p<.001	0.9144	p<.001	1.0635	p<.001
Age (≥85)	0.9247	p<.001	1.3825	p<.001	1.6545	p<.001
Sexe (Female)	0.1711	p<.001	0.2925	p<.001	0.2078	p<.001
Education (High)	-0.1343	p<.001	-0.1168	p<.001	-0.1400	p<.001
Education (Middle)	-0.1123	p<.001	-0.0805	p<.001	-0.1068	p<.001
Education (Student)	-0.3719	p<.001	-0.3435	p<.001	-0.1412	p<.01
Marital_Status (Married)	-0.1127	p<.001	-0.0603	p<.01	0.1479	p<.001
Employment (Employed)	-0.1632	p<.001	-0.1763	p<.001	0.2313	p<.001
Insurance (CMU-C)	-0.0803	p<.1	0.1682	p<.001	-0.1316	p<.001
Insurance (No_CS)	-0.1894	p<.05	0.0210	p<.1	-0.0549	p<.1
Income (<1500)	0.0679	p<.05	0.1452	p<.001	0.1157	p<.001
BMI_Status (Normal_weight)	-0.0972	p<.01	-0.0858	p<.001	-0.1634	p<.001
BMI_Status (Obese)	0.1972	p<.001	0.2031	p<.001	0.2097	p<.001
Smoking (Daily)	-0.0680	p<.1	0.1015	p<.001	0.1863	p<.001
Smoking (Occasional)	-0.0342	p<.1	0.1083	p<.01	-0.0414	p<.1
Drinking (Risky)	-0.0673	p<.1	0.0933	p<.001	-0.0399	p<.1
Nutrition (Insufficient)	-0.0882	p<.05	0.0538	p<.01	0.1894	p<.001
Health_Index (Good health)	-0.8944	p<.001	-		0.2407	p<.001
Health_Index (Moderate health)	-0.5349	p<.001	-		0.2532	p<.001
Health_Index (Fair health)	-0.3199	p<.001	-		0.1718	p<.001
Parents_Education (Non-response)	-		-0.3195	p<.001	-	
Parents_Education (With school)	-		-0.4224	p<.001	-	
Attitude_Future (Not preoccupied)	-		0.0772	p<.001	-	
Attitude_Future (Non-response)	-		0.2334	p<.001	-	
Member_Association (No)	-		-		-0.2848	p<.001

Notes: (1) The coefficients indicate the difference between the probit index (Z-score) of each category of each independent variable and that corresponding to the reference category of each independent variable. (2) The reference categories for the independent variables are: *low active* (physical activity); *15-24 years* (age group for those younger than 65 years old); *65-69 years* (age group for those of 65 years and older); *male* (sex of respondent); *primary education level* (education); *unmarried* (legal marital status); *unemployed* (employment status); *private health insurance* (insurance); *more than 1500 euros* (income level); *overweight* (BMI status); *never* (smoking); *no-risk* (alcohol consumption risk profile); *recommended* (nutrition – fruits and vegetables consumption); *poor health* (health index). (3) The reference categories for each instrumental variable are: *without school* (parents' education); *preoccupied* (attitude towards future); *yes* (membership in an association).

Table A11 Other healthcare services utilization

Variables	Prescribed medicines		Overnight hospitalization		Day hospitalization		Specialist physician visits		Preventive services	
Intercept	0.5470	p<.001	-0.4538	p<.001	-0.4545	p<.001	0.2804	p<.001	-0.1799	p<.1
PA (Moderate)	-0.0658	p<.05	-0.1095	p<.01	-0.0149	p<.1	-0.0343	p<.1	0.0719	p<.01
PA (High)	-0.1064	p<.001	-0.1296	p<.001	-0.0714	p<.1	-0.0415	p<.1	0.0826	p<.01
Age (25-44)	0.0123	p<.1	0.0154	p<.1	0.0610	p<.1	0.0257	p<.1	0.0371	p<.1
Age (45-64)	0.2670	p<.001	0.2639	p<.001	0.3021	p<.001	0.2047	p<.01	0.2850	p<.001
Age (65-74)	0.3344	p<.001	0.2639	p<.001	0.5306	p<.001	0.3463	p<.001	0.3417	p<.001
Age (75-84)	0.4449	p<.001	0.4443	p<.001	0.6412	p<.001	0.5364	p<.001	0.4730	p<.001
Age (≥85)	0.9083	p<.001	0.9037	p<.001	0.8374	p<.001	0.9359	p<.001	0.9254	p<.001
Sexe (Female)	0.1331	p<.001	0.0090	p<.1	-0.0724	p<.01	0.2378	p<.001	0.1649	p<.001
Education (High)	-0.0454	p<.1	0.0652	p<.1	0.0987	p<.01	0.2068	p<.001	-0.0541	p<.1
Education (Middle)	-0.0210	p<.1	0.0742	p<.05	0.1167	p<.001	0.0557	p<.05	-0.0086	p<.1
Education (Student)	-0.2794	p<.001	-0.4509	p<.001	0.1990	p<.01	-0.0892	p<.1	-0.4344	p<.001
Marital_Status (Married)	0.0449	p<.1	-0.0270	p<.1	-0.0204	p<.1	0.0426	p<.1	0.0845	p<.001
Employment (Employed)	-0.2567	p<.001	-0.1948	p<.001	-0.0813	p<.01	-0.1026	p<.001	-0.2132	p<.001
Insurance (CMU-C)	-0.0040	p<.1	0.0290	p<.1	0.0897	p<.1	-0.0358	p<.1	-0.0328	p<.1
Insurance (No_CS)	-0.3594	p<.001	-0.2752	p<.001	-0.1154	p<.1	-0.3076	p<.001	-0.3642	p<.001
Income (<1500)	-0.0693	p<.01	-0.0044	p<.1	-0.0633	p<.05	-0.1459	p<.001	-0.1092	p<.001
BMI_Status (Normal_weight)	-0.1454	p<.001	-0.0551	p<.1	-0.0147	p<.1	0.0382	p<.1	-0.1557	p<.001
BMI_Status (Obese)	0.2091	p<.001	-0.0090	p<.1	0.0324	p<.1	0.0161	p<.1	0.1520	p<.001
Smoking (Daily)	-0.0650	p<.01	-0.0835	p<.05	0.0793	p<.05	-0.1851	p<.001	-0.1467	p<.001
Smoking (Occasional)	-0.0818	p<.1	-0.0414	p<.1	-0.0612	p<.1	-0.0111	p<.1	0.0125	p<.1
Drinking (Risky)	-0.0838	p<.001	-0.1489	p<.001	-0.0505	p<.1	-0.0644	p<.01	-0.1150	p<.001
Nutrition (Insufficient)	-0.1092	p<.001	-0.0307	p<.1	0.0033	p<.1	-0.1131	p<.001	-0.1607	p<.001
Health_Index (Good health)	-0.8782	p<.001	-0.6898	p<.001	-0.6609	p<.001	-0.8155	p<.001	-0.5204	p<.001
Health_Index (Moderate health)	-0.6192	p<.001	-0.4327	p<.001	-0.3399	p<.001	-0.5914	p<.001	-0.3972	p<.001
Health_Index (Fair health)	-0.3030	p<.001	-0.3800	p<.001	-0.1281	p<.001	-0.2878	p<.001	-0.2665	p<.001

Notes: (1) The coefficients indicate the difference between the probit index (Z-score) of each category of each independent variable and that corresponding to the reference category of each independent variable. (2) The reference categories for the independent variables are: *low active* (physical activity); *15-24 years* (age group for those younger than 65 years old); *65-69 years* (age group for those of 65 years and older); *male* (sex of respondent); *primary education level* (education); *unmarried* (legal marital status); *unemployed* (employment status); *private health insurance* (insurance); *more than 1500 euros* (income level); *overweight* (BMI status); *never* (smoking); *no-risk* (alcohol consumption risk profile); *recommended* (nutrition – fruits and vegetables consumption); *poor health* (health index).

Table A12 Non-prescribed medicines

Variables	< 65 years old						≥65 years old	
	Model 1						Model 2	
	Non-prescribed medicines		Health Index		Physical activity		Non-prescribed medicines	
Intercept	-1.6031	p<.001	0.5851	p<.001	0.2450	p<.001	-0.6442	p<.001
PA (Moderate)	0.1146	p<.001	0.1633	p<.001	-		0.0804	p<.1
PA (High)	0.1528	p<.001	0.1177	p<.001	-		0.1853	p<.01
Age (25-34)	0.3438	p<.001	0.3171	p<.001	0.1929	p<.001	-	
Age (35-44)	0.4252	p<.001	0.4197	p<.001	0.2505	p<.001	-	
Age (45-54)	0.4477	p<.001	0.5555	p<.001	0.2718	p<.001	-	
Age (55-64)	0.3658	p<.001	0.6226	p<.001	0.0793	p<.1	-	
Age (70-74)	-		-		-		-0.0531	p<.1
Age (75-79)	-		-		-		-0.1707	p<.01
Age (80-older)	-		-		-		-0.3182	p<.001
Sexe (Female)	0.3899	p<.001	0.2862	p<.001	0.1897	p<.001	0.0644	p<.1
Education (High)	0.3252	p<.001	-0.1324	p<.001	-0.1461	p<.001	0.1560	p<.1
Education (Middle)	0.2013	p<.001	-0.0596	p<.01	-0.0939	p<.001	0.1027	p<.1
Education (Student)	0.2679	p<.001	-0.0776	p<.1	-0.3653	p<.001	-	
Marital_Status (Married)	-0.0557	p<.1	-0.1062	p<.001	0.1505	p<.001	-0.0191	p<.1
Employment (Employed)	-0.0529	p<.1	-0.1818	p<.001	0.1398	p<.001	0.1037	p<.1
Insurance (CMU-C)	0.0580	p<.001	0.1788	p<.001	-0.1691	p<.001	0.0574	p<.1
Insurance (No_CS)	0.0907	p<.1	-0.0110	p<.1	-0.0844	p<.1	0.0878	p<.1
Income (<1500)	-0.0335	p<.1	0.1225	p<.001	0.0976	p<.001	-0.0505	p<.05
BMI_Status (Normal_weight)	0.0610	p<.05	-0.0583	p<.01	-0.1744	p<.001	0.1247	p<.05
BMI_Status (Obese)	-0.0249	p<.1	0.2371	p<.001	0.1610	p<.001	-0.0803	p<.1
Smoking (Daily)	0.0135	p<.1	0.1176	p<.001	0.2158	p<.001	-0.2790	p<.01
Smoking (Occasional)	0.1391	p<.01	0.1187	p<.01	-0.0114	p<.1	-0.0374	p<.1
Drinking (Risky)	0.1040	p<.001	0.0999	p<.001	-0.0374	p<.1	-0.1574	p<.05
Nutrition (Insufficient)	0.1859	p<.001	0.0520	p<.05	0.1748	p<.001	-0.0143	p<.1
Health_Index (Good health)	-0.6648	p<.001	-		0.1704	p<.001	-0.6484	p<.001
Health_Index (Moderate health)	-0.2568	p<.001	-		0.1526	p<.001	-0.2279	p<.001
Health_Index (Fair health)	-0.1512	p<.001	-		0.0990	p<.001	-0.3418	p<.001
Parents_Education (Non-response)	-		-0.2910	p<.001	-		-	
Parents_Education (With school)	-		-0.4179	p<.001	-		-	
Attitude_Future (Not preoccupied)	-		0.0596	p<.001	-		-	
Attitude_Future (Non-response)	-		0.2035	p<.01	-		-	
Member_Association (No)	-		-		-0.2805	p<.001	-	

Notes: (1) The coefficients indicate the difference between the probit index (Z-score) of each category of each independent variable and that corresponding to the reference category of each independent variable. (2) The reference categories for the independent variables are: *low active* (physical activity); *15-24 years* (age group for those younger than 65 years old); *65-69 years* (age group for those of 65 years and older); *male* (sex of respondent); *primary education level* (education); *unmarried* (legal marital status); *unemployed* (employment status); *private health insurance* (insurance); *more than 1500 euros* (income level); *overweight* (BMI status); *never* (smoking); *no-risk* (alcohol consumption risk profile); *recommended* (nutrition – fruits and vegetables consumption); *poor health* (health index). (3) The reference categories for each instrumental variable are: *without school* (parents' education); *preoccupied* (attitude towards future); *yes* (membership in an association).

Table A13 Prescribed medication

Variables	< 65 years old Model 1		≥65 years old Model 2	
Intercept	0.1440	p<.05	1.4719	p<.001
PA (Moderate)	-0.0261	p<.1	-0.1349	p<.1
PA (High)	-0.1006	p<.001	-0.1273	p<.1
Age (25-34)	0.1588	p<.01	-	
Age (35-44)	0.2616	p<.001	-	
Age (45-54)	0.4735	p<.001	-	
Age (55-64)	0.8371	p<.001	-	
Age (70-74)	-		0.3617	p<.001
Age (75-79)	-		0.4637	p<.001
Age (80-older)	-		0.6107	p<.001
Sexe (Female)	0.1748	p<.001	-0.0706	p<.1
Education (High)	-0.0964	p<.01	0.1168	p<.1
Education (Middle)	-0.0426	p<.1	0.1083	p<.1
Education (Student)	-0.0368	p<.1	-	
Marital_Status (Married)	0.0174	p<.1	-0.0984	p<.1
Employment (Employed)	-0.1691	p<.001	-0.0447	p<.1
Insurance (CMU-C)	0.0225	p<.1	-0.1138	p<.1
Insurance (No_CS)	-0.3810	p<.001	-0.1312	p<.1
Income (<1500)	-0.0371	p<.1	-0.1259	p<.05
BMI_Status (Normal_weight)	-0.1069	p<.001	-0.2421	p<.001
BMI_Status (Obese)	0.2127	p<.001	0.1861	p<.01
Smoking (Daily)	-0.0576	p<.05	-0.1199	p<.1
Smoking (Occasional)	-0.0855	p<.1	0.1209	p<.1
Drinking (Risky)	-0.0797	p<.01	-0.0384	p<.1
Nutrition (Inssuficient)	-0.0875	p<.01	-0.1114	p<.1
Health_Index (Good health)	-0.9720	p<.001	-0.9812	p<.001
Health_Index (Moderate health)	-0.6987	p<.001	-0.6083	p<.001
Health_Index (Fair health)	-0.3578	p<.001	-0.3083	p<.001

Notes: (1) The coefficients indicate the difference between the probit index (Z-score) of each category of each independent variable and that corresponding to the reference category of each independent variable. (2) The reference categories for the independent variables are: *low active* (physical activity); *15-24 years* (age group for those younger than 65 years old); *65-69 years* (age group for those of 65 years and older); *male* (sex of respondent); *primary education level* (education); *unmarried* (legal marital status); *unemployed* (employment status); *private health insurance* (insurance); *more than 1500 euros* (income level); *overweight* (BMI status); *never* (smoking); *no-risk* (alcohol consumption risk profile); *recommended* (nutrition – fruits and vegetables consumption); *poor health* (health index). (3) The reference categories for each instrumental variable are: *without school* (parents' education); *preoccupied* (attitude towards future); *yes* (membership in an association).

Table A14 Overnight hospitalization

	< 65 years old						≥65 years old	
	Model 1						Model 2	
**Variables**	**Overnight hospitalization**		**Health Index**		**Physical activity**		**Overnight hospitalization**	
Intercept	-0.5294	p<.001	0.5518	p<.001	0.2367	p<.001	-0.2876	p<.01
PA (Moderate)	-0.2084	p<.001	0.1613	p<.001	-		-0.0849	p<.1
PA (High)	-0.4232	p<.001	0.1187	p<.001	-		-0.1124	p<.001
Age (25-34)	0.0037	p<.1	0.3210	p<.001	0.1853	p<.001	-	
Age (35-44)	0.3040	p<.001	0.4223	p<.001	0.2450	p<.001	-	
Age (45-54)	0.1884	p<.01	0.5524	p<.001	0.2712	p<.001	-	
Age (55-64)	0.1573	p<.01	0.6218	p<.001	0.0732	p<.1	-	
Age (70-74)	-		-		-		0.0551	p<.1
Age (75-79)	-		-		-		-0.0395	p<.1
Age (80-older)	-		-		-		-0.1169	p<.1
Sexe (Female)	0.1365	p<.001	0.2851	p<.001	0.1943	p<.001	-0.3594	p<.001
Education (High)	0.1244	p<.001	-0.1434	p<.001	-0.1370	p<.001	0.0962	p<.1
Education (Middle)	0.1047	p<.01	-0.0650	p<.01	-0.0904	p<.001	0.0139	p<.1
Education (Student)	-0.1257	p<.001	-0.0799	p<.1	-0.3602	p<.001	-	
Marital_Status (Married)	-0.0430	p<.1	-0.1028	p<.001	0.1549	p<.001	-0.0566	p<.1
Employment (Employed)	-0.1908	p<.001	-0.1818	p<.001	0.1354	p<.001	0.0070	p<.1
Insurance (CMU-C)	-0.0604	p<.1	0.1763	p<.001	-0.1699	p<.001	-0.0514	p<.1
Insurance (No_CS)	-0.4356	p<.001	-0.0042	p<.1	-0.0817	p<.1	0.0186	p<.1
Income (<1500)	0.0428	p<.1	0.1273	p<.001	0.0959	p<.001	-0.0419	p<.1
BMI_Status (Normal_weight)	-0.1299	p<.001	-0.0606	p<.01	-0.1735	p<.001	0.1465	p<.01
BMI_Status (Obese)	-0.0442	p<.1	0.2336	p<.001	0.1663	p<.001	0.0686	p<.1
Smoking (Daily)	-0.0578	p<.1	0.1127	p<.001	0.2227	p<.001	-0.1916	p<.1
Smoking (Occasional)	-0.0180	p<.1	0.1140	p<.01	-0.0090	p<.1	-0.2083	p<.1
Drinking (Risky)	-0.1589	p<.001	0.1007	p<.001	-0.0343	p<.1	-0.0809	p<.1
Nutrition (Insufficient)	-0.0375	p<.1	0.0531	p<.05	0.1773	p<.001	0.0129	p<.1
Health_Index (Good health)	-0.6933	p<.001	-		0.1642	p<.001	-0.5734	p<.001
Health_Index (Moderate health)	-0.3075	p<.001	-		0.1507	p<.001	-0.5717	p<.001
Health_Index (Fair health)	-0.0955	p<.01	-		0.0950	p<.01	-0.4021	p<.001
Parents_Education (Non-response)	-		-0.2642	p<.001	-		-	
Parents_Education (With school)	-		-0.3858	p<.001	-		-	
Attitude_Future (Not preoccupied)	-		0.0539	p<.01	-		-	
Attitude_Future (Non-response)	-		0.2636	p<.001	-		-	
Member_Association (No)	-		-		-0.2834	p<.001	-	

Notes: (1) The coefficients indicate the difference between the probit index (Z-score) of each category of each independent variable and that corresponding to the reference category of each independent variable. (2) The reference categories for the independent variables are: *low active* (physical activity); *15-24 years* (age group for those younger than 65 years old); *65-69 years* (age group for those of 65 years and older); *male* (sex of respondent); *primary education level* (education); *unmarried* (legal marital status); *unemployed* (employment status); *private health insurance* (insurance); *more than 1500 euros* (income level); *overweight* (BMI status); *never* (smoking); *no-risk* (alcohol consumption risk profile); *recommended* (nutrition – fruits and vegetables consumption); *poor health* (health index). (3) The reference categories for each instrumental variable are: *without school* (parents' education); *preoccupied* (attitude towards future); *yes* (membership in an association).

Table A15 Day hospitalization

Variables	< 65 years old						≥65 years old	
	Model 1						Model 2	
	Day hospitalization		Health Index		Physical activity		Day hospitalization	
Intercept	-0.7210	p<.05	0.5429	p<.001	0.2395	p<.001	-0.5896	p<.001
PA (Moderate)	-0.0811	p<.01	0.1631	p<.001	-		0.0387	p<.1
PA (High)	-0.2783	p<.001	0.1198	p<.001	-		0.0484	p<.1
Age (25-34)	-0.1215	p<.1	0.3201	p<.001	0.1879	p<.001	-	
Age (35-44)	-0.2035	p<.1	0.4173	p<.001	0.2480	p<.001	-	
Age (45-54)	-0.1310	p<.1	0.5517	p<.001	0.2763	p<.001	-	
Age (55-64)	-0.1917	p<.1	0.6140	p<.001	0.0757	p<.1	-	
Age (70-74)	-		-		-		-0.0117	.
Age (75-79)	-		-		-		-0.0721	.
Age (80-older)	-		-		-		-0.2545	p<.001
Sexe (Female)	0.0656	p<.1	0.2853	p<.001	0.1963	p<.001	-0.2630	p<.001
Education (High)	0.0361	p<.1	-0.1411	p<.001	-0.1346	p<.001	0.1912	p<.05
Education (Middle)	0.0546	p<.1	-0.0625	p<.01	-0.0920	p<.001	0.2792	p<.001
Education (Student)	-0.0308	p<.1	-0.0832	.	-0.3621	p<.001	-	
Marital_Status (Married)	-0.0265	p<.1	-0.1003	p<.001	0.1551	p<.001	0.0140	p<.1
Employment (Employed)	-0.1402	p<.001	-0.1845	p<.001	0.1338	p<.001	0.2886	p<.1
Insurance (CMU-C)	0.1470	p<.01	0.1730	p<.001	-0.1612	p<.001	-0.0801	p<.1
Insurance (No_CS)	-0.2444	p<.01	0.0005	p<.1	-0.0820	p<.1	0.0935	p<.1
Income (<1500)	-0.0375	p<.1	0.1245	p<.001	0.0927	p<.001	-0.0198	p<.1
BMI_Status (Normal_weight)	-0.0624	p<.1	-0.0578	p<.01	-0.1734	p<.001	0.0250	p<.1
BMI_Status (Obese)	0.0875	p<.1	0.2400	p<.001	0.1581	p<.001	-0.0116	p<.1
Smoking (Daily)	0.1025	p<.01	0.1068	p<.001	0.2257	p<.001	-0.1918	p<.1
Smoking (Occasional)	-0.0624	p<.1	0.1143	p<.01	-0.0106	p<.1	-0.0208	p<.1
Drinking (Risky)	-0.0289	p<.1	0.1004	p<.001	-0.0369	p<.1	-0.0725	p<.1
Nutrition (Insufficient)	0.0032	p<.1	0.0546	p<.05	0.1815	p<.001	-0.0041	p<.1
Health_Index (Good health)	-0.4263	p<.1	-		0.1678	p<.001	-0.5141	p<.001
Health_Index (Moderate health)	-0.2518	p<.1	-		0.1544	p<.001	-0.3118	p<.001
Health_Index (Fair health)	-0.1074	p<.1	-		0.0997	p<.001	-0.2156	p<.001
Parents_Education (Non-response)	-		-0.2458	p<.001	-		-	
Parents_Education (With school)	-		-0.3702	p<.001	-		-	
Attitude_Future (Not preoccupied)	-		0.0573	p<.01	-		-	
Attitude_Future (Non-response)	-		0.2488	p<.001	-		-	
Member_Association (No)	-		-		-0.2834	p<.001	-	

Notes: (1) The coefficients indicate the difference between the probit index (Z-score) of each category of each independent variable and that corresponding to the reference category of each independent variable. (2) The reference categories for the independent variables are: *low active* (physical activity); *15-24 years* (age group for those younger than 65 years old); *65-69 years* (age group for those of 65 years and older); *male* (sex of respondent); *primary education level* (education); *unmarried* (legal marital status); *unemployed* (employment status); *private health insurance* (insurance); *more than 1500 euros* (income level); *overweight* (BMI status); *never* (smoking); *no-risk* (alcohol consumption risk profile); *recommended* (nutrition – fruits and vegetables consumption); *poor health* (health index). (3) The reference categories for each instrumental variable are: *without school* (parents' education); *preoccupied* (attitude towards future); *yes* (membership in an association).

Table A16a Generalist physician visits (Model 1)

Variables	< 65 years old					
	Generalist visits		Health Index		Physical activity	
Intercept	1.9823	p<.001	0.5682	p<.001	0.2413	p<.001
PA (Moderate)	0.1917	p<.001	0.1588	p<.001	-	
PA (High)	0.1802	p<.001	0.1160	p<.001	-	
Age (25-34)	0.1732	p<.01	0.3223	p<.001	0.1867	p<.001
Age (35-44)	0.1899	p<.01	0.4216	p<.001	0.2504	p<.001
Age (45-54)	0.2201	p<.01	0.5560	p<.001	0.2733	p<.001
Age (55-64)	0.2049	p<.05	0.6182	p<.001	0.0723	p<.1
Sexe (Female)	0.1848	p<.001	0.2833	p<.001	0.1907	p<.001
Education (High)	-0.0281	p<.1	-0.1397	p<.001	-0.1398	p<.001
Education (Middle)	-0.0074	p<.1	-0.0634	p<.01	-0.0927	p<.001
Education (Student)	0.1940	p<.01	-0.0884	p<.1	-0.3668	p<.001
Marital_Status (Married)	0.1770	p<.001	-0.1066	p<.001	0.1520	p<.001
Employment (Employed)	0.0463	p<.1	-0.1875	p<.001	0.1340	p<.001
Insurance (CMU-C)	-0.0310	p<.1	0.1781	p<.001	-0.1673	p<.001
Insurance (No_CS)	-0.4714	p<.001	-0.0060	p<.1	-0.0810	p<.1
Income (<1500)	-0.0955	p<.01	0.1278	p<.001	0.0959	p<.001
BMI_Status (Normal_weight)	-0.0420	p<.1	-0.0629	p<.01	-0.1711	p<.001
BMI_Status (Obese)	-0.0353	p<.1	0.2358	p<.001	0.1631	p<.001
Smoking (Daily)	-0.1827	p<.001	0.1145	p<.001	0.2194	p<.001
Smoking (Occasional)	-0.0702	p<.1	0.1128	p<.01	-0.0078	p<.1
Drinking (Risky)	-0.1117	p<.001	0.1005	p<.001	-0.0347	p<.1
Nutrition (Insufficient)	-0.0626	p<.1	0.0529	p<.05	0.1744	p<.001
Health_Index (Good health)	-0.6639	p<.001	-		0.1631	p<.001
Health_Index (Moderate health)	-0.4507	p<.001	-		0.1496	p<.001
Health_Index (Fair health)	-0.2705	p<.001	-		0.0936	p<.01
Parents_Education (Non-response)	-		-0.2731	p<.001	-	
Parents_Education (With school)	-		-0.3898	p<.001	-	
Attitude_Future (Not preoccupied)	-		0.0655	p<.001	-	
Attitude_Future (Non-response)	-		0.2852	p<.001	-	
Member_Association (No)	-		-		-0.2846	p<.001

Notes: (1) The coefficients indicate the difference between the probit index (Z-score) of each category of each independent variable and that corresponding to the reference category of each independent variable. (2) The reference categories for the independent variables are: *low active* (physical activity); *15-24 years* (age group for those younger than 65 years old); *65-69 years* (age group for those of 65 years and older); *male* (sex of respondent); *primary education level* (education); *unmarried* (legal marital status); *unemployed* (employment status); *private health insurance* (insurance); *more than 1500 euros* (income level); *overweight* (BMI status); *never* (smoking); *no-risk* (alcohol consumption risk profile); *recommended* (nutrition – fruits and vegetables consumption); *poor health* (health index). (3) The reference categories for each instrumental variable are: *without school* (parents' education); *preoccupied* (attitude towards future); *yes* (membership in an association).

Table A16b Generalist physician visits (Model 2)

Variables	≥ 65 years old					
	Generalist visits		Health Index		Physical activity	
Intercept	2.9791	p<.001	1.7380	p<.001	0.4214	p<.001
PA (Moderate)	0.2615	p<.01	0.5113	p<.001	-	
PA (High)	0.2613	p<.01	0.3480	p<.001	-	
Age (70-74)	0.0841	p<.1	0.2577	p<.001	-0.0492	p<.1
Age (75-79)	0.0779	p<.1	0.4939	p<.001	0.0720	p<.1
Age (80-older)	-0.1343	p<.1	0.6837	p<.001	0.4597	p<.001
Sexe (Female)	-0.1951	p<.05	0.2750	p<.001	0.2537	p<.001
Education (High)	0.3359	p<.01	-0.1290	.	-0.1470	p<.05
Education (Middle)	0.3268	p<.01	-0.1411	p<.01	-0.1605	p<.01
Marital_Status (Married)	-0.2874	p<.01	-0.0989	p<.05	0.0518	p<.1
Employment (Employed)	0.0881	p<.1	-0.2344	.	0.2570	p<.1
Insurance (CMU-C)	0.0028	p<.1	0.5207	p<.01	-0.1475	p<.1
Insurance (No_CS)	-0.6548	p<.001	0.1346	.	0.1005	p<.1
Income (<1500)	-0.2683	p<.01	0.1882	p<.001	0.0648	p<.1
BMI_Status (Normal_weight)	-0.1371	p<.1	-0.0916	p<.05	-0.1451	p<.001
BMI_Status (Obese)	0.2975	p<.05	0.1133	p<.05	0.3513	p<.001
Smoking (Daily)	-0.3924	p<.01	0.0040	.	-0.0895	p<.1
Smoking (Occasional)	-0.6312	p<.001	-0.0242	.	-0.2186	p<.1
Drinking (Risky)	0.0432	p<.1	0.0764	.	0.0236	p<.1
Nutrition (Insufficient)	-0.2491	p<.1	0.2493	p<.001	0.2875	p<.001
Health_Index (Good health)	-0.9956	p<.001	-		0.3713	p<.001
Health_Index (Moderate health)	-0.6640	p<.001	-		0.5285	p<.001
Health_Index (Fair health)	-0.4988	p<.001	-		0.3000	p<.001
Parents_Education (Non-response)	-		-0.5289	p<.001	-	
Parents_Education (With school)	-		-0.5524	p<.001	-	
Attitude_Future (Not preoccupied)	-		0.2181	p<.001	-	
Attitude_Future (Non-response)	-		0.3677	p<.01	-	
Member_Association (No)	-		-		-0.3083	p<.001

Notes: (1) The coefficients indicate the difference between the probit index (Z-score) of each category of each independent variable and that corresponding to the reference category of each independent variable. (2) The reference categories for the independent variables are: *low active* (physical activity); *15-24 years* (age group for those younger than 65 years old); *65-69 years* (age group for those of 65 years and older); *male* (sex of respondent); *primary education level* (education); *unmarried* (legal marital status); *unemployed* (employment status); *private health insurance* (insurance); *more than 1500 euros* (income level); *overweight* (BMI status); *never* (smoking); *no-risk* (alcohol consumption risk profile); *recommended* (nutrition – fruits and vegetables consumption); *poor health* (health index). (3) The reference categories for each instrumental variable are: *without school* (parents' education); *preoccupied* (attitude towards future); *yes* (membership in an association).

Table A17 Specialist physician visits

Variables	< 65 years old		≥65 years old	
	Model 1		Model 2	
Intercept	0.1119	p<.1	0.6018	p<.001
PA (Moderate)	0.0430	p<.1	0.0019	p<.1
PA (High)	0.0482	p<.1	0.0385	p<.1
Age (25-34)	0.2618	p<.001	-	
Age (35-44)	0.2122	p<.001	-	
Age (45-54)	0.2877	p<.001	-	
Age (55-64)	0.2522	p<.001	-	
Age (70-74)	-		0.1207	p<.05
Age (75-79)	-		0.0461	p<.1
Age (80-older)	-		-0.0714	p<.1
Sexe (Female)	0.3291	p<.001	-0.1196	p<.01
Education (High)	0.1735	p<.001	0.1595	p<.05
Education (Middle)	0.1285	p<.001	0.1449	p<.05
Education (Student)	0.0306	p<.1	-	
Marital_Status (Married)	0.0012	p<.1	0.0157	p<.1
Employment (Employed)	-0.1215	p<.001	-0.0583	p<.1
Insurance (CMU-C)	-0.0731	p<.1	0.2901	p<.1
Insurance (No_CS)	-0.3760	p<.001	-0.0898	p<.1
Income (<1500)	-0.1596	p<.001	-0.0963	p<.05
BMI_Status (Normal_weight)	0.0303	p<.1	0.0960	p<.1
BMI_Status (Obese)	0.0189	p<.1	0.0043	p<.1
Smoking (Daily)	-0.1776	p<.001	-0.2532	p<.01
Smoking (Occasional)	0.0105	p<.1	-0.2730	p<.1
Drinking (Risky)	-0.0856	p<.001	0.1096	p<.1
Nutrition (Inssuficient)	-0.0865	p<.001	-0.2215	p<.01
Health_Index (Good health)	-0.8054	p<.001	-0.7950	p<.001
Health_Index (Moderate health)	-0.5727	p<.001	-0.6782	p<.001
Health_Index (Fair health)	-0.2842	p<.001	-0.3048	p<.01

Notes: (1) The coefficients indicate the difference between the probit index (Z-score) of each category of each independent variable and that corresponding to the reference category of each independent variable. (2) The reference categories for the independent variables are: *low active* (physical activity); *15-24 years* (age group for those younger than 65 years old); *65-69 years* (age group for those of 65 years and older); *male* (sex of respondent); *primary education level* (education); *unmarried* (legal marital status); *unemployed* (employment status); *private health insurance* (insurance); *more than 1500 euros* (income level); *overweight* (BMI status); *never* (smoking); *no-risk* (alcohol consumption risk profile); *recommended* (nutrition – fruits and vegetables consumption); *poor health* (health index).

Table A18 Preventive services

Variables	< 65 years old Model 1		≥65 years old Model 2	
Intercept	-0.2147	p<.01	1.4490	p<.001
PA (Moderate)	0.0851	p<.01	-0.0343	p<.1
PA (High)	0.0880	p<.01	-0.0683	p<.1
Age (25-34)	0.2438	p<.001	-	
Age (35-44)	0.2798	p<.001	-	
Age (45-54)	0.6222	p<.001	-	
Age (55-64)	0.9845	p<.001	-	
Age (70-74)	-		0.1812	p<.05
Age (75-79)	-		0.3146	p<.001
Age (80-older)	-		0.4006	p<.001
Sexe (Female)	0.2252	p<.001	-0.1237	p<.05
Education (High)	-0.0679	p<.1	-0.0816	p<.1
Education (Middle)	-0.0055	p<.1	-0.0058	p<.1
Education (Student)	-0.2986	p<.001	-	
Marital_Status (Married)	0.0509	p<.1	-0.0230	p<.1
Employment (Employed)	-0.1244	p<.001	-0.2471	p<.1
Insurance (CMU-C)	-0.0207	p<.1	0.1445	p<.1
Insurance (No_CS)	-0.3762	p<.001	-0.2128	p<.1
Income (<1500)	-0.0741	p<.01	-0.1735	p<.01
BMI_Status (Normal_weight)	0.1655	p<.001	0.1606	p<.01
BMI_Status (Obese)	-0.1595	p<.001	-0.1698	p<.05
Smoking (Daily)	-0.1206	p<.001	-0.2180	p<.05
Smoking (Occasional)	0.0354	p<.1	0.0064	p<.1
Drinking (Risky)	-0.1134	p<.001	-0.0183	p<.1
Nutrition (Inssuficient)	-0.1297	p<.001	-0.2820	p<.001
Health_Index (Good health)	-0.4836	p<.001	-0.6037	p<.001
Health_Index (Moderate health)	-0.3770	p<.001	-0.4397	p<.001
Health_Index (Fair health)	-0.2499	p<.001	-0.3276	p<.001

Notes: (1) The coefficients indicate the difference between the probit index (Z-score) of each category of each independent variable and that corresponding to the reference category of each independent variable. (2) The reference categories for the independent variables are: *low active* (physical activity); *15-24 years* (age group for those younger than 65 years old); *65-69 years* (age group for those of 65 years and older); *male* (sex of respondent); *primary education level* (education); *unmarried* (legal marital status); *unemployed* (employment status); *private health insurance* (insurance); *more than 1500 euros* (income level); *overweight* (BMI status); *never* (smoking); *no-risk* (alcohol consumption risk profile); *recommended* (nutrition – fruits and vegetables consumption); *poor health* (health index).

Table A19a Home healthcare services (Model 1)

Variables	< 65 years old					
	Home healthcare		Health Index		Physical activity	
Intercept	-1.8197	p<.001	0.5402	p<.001	0.2414	p<.001
PA (Moderate)	-0.1921	p<.001	0.1698	p<.001	-	
PA (High)	-0.2247	p<.001	0.1254	p<.001	-	
Age (25-34)	0.3719	p<.001	0.3304	p<.001	0.1864	p<.001
Age (35-44)	0.2946	p<.001	0.4272	p<.001	0.2463	p<.001
Age (45-54)	0.3621	p<.001	0.5696	p<.001	0.2722	p<.001
Age (55-64)	0.3312	p<.001	0.6302	p<.001	0.0754	p<.1
Sexe (Female)	0.1822	p<.001	0.2881	p<.001	0.1930	p<.001
Education (High)	-0.1737	p<.001	-0.1383	p<.001	-0.1420	p<.001
Education (Middle)	-0.0973	p<.01	-0.0657	p<.01	-0.0917	p<.001
Education (Student)	-0.2047	p<.01	-0.0837	p<.1	-0.3548	p<.001
Marital_Status (Married)	-0.1133	p<.001	-0.1076	p<.001	0.1507	p<.001
Employment (Employed)	-0.2133	p<.001	-0.1916	p<.001	0.1414	p<.001
Insurance (CMU-C)	0.0690	p<.1	0.1708	p<.001	-0.1609	p<.001
Insurance (No_CS)	-0.2665	p<.01	0.0102	p<.1	-0.0838	p<.1
Income (<1500)	0.0236	p<.1	0.1265	p<.001	0.0971	p<.001
BMI_Status (Normal_weight)	-0.1114	p<.001	-0.0615	p<.01	-0.1777	p<.001
BMI_Status (Obese)	0.2293	p<.001	0.2354	p<.001	0.1582	p<.001
Smoking (Daily)	0.0462	p<.1	0.1182	p<.001	0.2165	p<.001
Smoking (Occasional)	0.0945	p<.1	0.1142	p<.01	-0.0149	p<.1
Drinking (Risky)	-0.0394	p<.1	0.1047	p<.001	-0.0368	p<.1
Nutrition (Insufficient)	-0.0477	p<.1	0.0491	p<.05	0.1761	p<.001
Health_Index (Good health)	-0.8132	p<.001	-		0.1685	p<.001
Health_Index (Moderate health)	-0.5149	p<.001	-		0.1494	p<.001
Health_Index (Fair health)	-0.2486	p<.001	-		0.0957	p<.01
Parents_Education (Non-response)	-		-0.2300	p<.001	-	
Parents_Education (With school)	-		-0.3624	p<.001	-	
Attitude_Future (Not preoccupied)	-		0.0621	p<.001	-	
Attitude_Future (Non-response)	-		0.2322	p<.001	-	
Member_Association (No)	-		-		-0.2834	p<.001

Notes: (1) The coefficients indicate the difference between the probit index (Z-score) of each category of each independent variable and that corresponding to the reference category of each independent variable. (2) The reference categories for the independent variables are: *low active* (physical activity); *15-24 years* (age group for those younger than 65 years old); *65-69 years* (age group for those of 65 years and older); *male* (sex of respondent); *primary education level* (education); *unmarried* (legal marital status); *unemployed* (employment status); *private health insurance* (insurance); *more than 1500 euros* (income level); *overweight* (BMI status); *never* (smoking); *no-risk* (alcohol consumption risk profile); *recommended* (nutrition – fruits and vegetables consumption); *poor health* (health index). (3) The reference categories for each instrumental variable are: *without school* (parents' education); *preoccupied* (attitude towards future); *yes* (membership in an association).

Table A19b Home healthcare services (Model 2)

Variables	≥ 65 years old					
	Non-prescribed medicines		Health Index		Physical activity	
Intercept	-0.7496	p<.001	1.7219	p<.001	0.4187	p<.001
PA (Moderate)	-0.2599	p<.001	0.5123	p<.001	-	
PA (High)	-0.4213	p<.001	0.3410	p<.001	-	
Age (70-74)	0.0903	p<.1	0.2648	p<.001	-0.0660	p<.1
Age (75-79)	0.3643	p<.001	0.4887	p<.001	0.0708	p<.1
Age (80-older)	0.7830	p<.001	0.7007	p<.001	0.4483	p<.001
Sexe (Female)	0.0655	p<.1	0.2666	p<.001	0.2622	p<.001
Education (High)	-0.0832	p<.1	-0.1207	p<.1	-0.1554	p<.05
Education (Middle)	-0.1484	p<.1	-0.1252	p<.05	-0.1631	p<.01
Marital_Status (Married)	-0.2325	p<.001	-0.0974	p<.05	0.0489	p<.1
Employment (Employed)	-0.1861	p<.1	-0.2825	p<.1	0.3425	p<.05
Insurance (CMU-C)	0.0531	p<.1	0.5422	p<.01	-0.1642	p<.1
Insurance (No_CS)	0.0874	p<.1	0.1426	p<.1	0.1258	p<.1
Income (<1500)	0.1120	p<.1	0.1975	p<.001	0.0798	p<.1
BMI_Status (Normal_weight)	-0.0128	p<.1	-0.1019	p<.05	-0.1521	p<.001
BMI_Status (Obese)	0.1644	p<.05	0.1058	p<.1	0.3522	p<.001
Smoking (Daily)	-0.3274	p<.01	0.0167	p<.1	-0.0944	p<.1
Smoking (Occasional)	-0.3066	p<.1	-0.0283	p<.1	-0.2073	p<.1
Drinking (Risky)	-0.2014	p<.01	0.0650	p<.1	0.0353	p<.1
Nutrition (Insufficient)	0.1140	p<.1	0.2477	p<.001	0.2911	p<.001
Health_Index (Good health)	-0.5348	p<.001	-		0.3639	p<.001
Health_Index (Moderate health)	-0.6436	p<.001	-		0.5326	p<.001
Health_Index (Fair health)	-0.4631	p<.001	-		0.2994	p<.001
Parents_Education (Non-response)	-		-0.5153	p<.001	-	
Parents_Education (With school)	-		-0.5520	p<.001	-	
Attitude_Future (Not preoccupied)	-		0.2000	p<.001	-	
Attitude_Future (Non-response)	-		0.2963	p<.001	-	
Member_Association (No)	-		-		-0.3000	p<.001

Notes: (1) The coefficients indicate the difference between the probit index (Z-score) of each category of each independent variable and that corresponding to the reference category of each independent variable. (2) The reference categories for the independent variables are: *low active* (physical activity); *15-24 years* (age group for those younger than 65 years old); *65-69 years* (age group for those of 65 years and older); *male* (sex of respondent); *primary education level* (education); *unmarried* (legal marital status); *unemployed* (employment status); *private health insurance* (insurance); *more than 1500 euros* (income level); *overweight* (BMI status); *never* (smoking); *no-risk* (alcohol consumption risk profile); *recommended* (nutrition – fruits and vegetables consumption); *poor health* (health index). (3) The reference categories for each instrumental variable are: *without school* (parents' education); *preoccupied* (attitude towards future); *yes* (membership in an association).
